# Non-antibiotic pharmaceuticals enhance the transmission of exogenous antibiotic resistance genes through bacterial transformation

**DOI:** 10.1038/s41396-020-0679-2

**Published:** 2020-05-18

**Authors:** Yue Wang, Ji Lu, Jan Engelstädter, Shuai Zhang, Pengbo Ding, Likai Mao, Zhiguo Yuan, Philip L. Bond, Jianhua Guo

**Affiliations:** 1 Advanced Water Management Centre, The University of Queensland, Brisbane, QLD 4072 Australia; 2 School of Biological Sciences, The University of Queensland, Brisbane, QLD 4072 Australia

**Keywords:** Antibiotics, Next-generation sequencing

## Abstract

Antibiotic resistance is a serious global threat for public health. Considering the high abundance of cell-free DNA encoding antibiotic resistance genes (ARGs) in both clinical and environmental settings, natural transformation is an important horizontal gene transfer pathway to transmit antibiotic resistance. It is acknowledged that antibiotics are key drivers for disseminating antibiotic resistance, yet the contributions of non-antibiotic pharmaceuticals on transformation of ARGs are overlooked. In this study, we report that some commonly consumed non-antibiotic pharmaceuticals, at clinically and environmentally relevant concentrations, significantly facilitated the spread of antibiotic resistance through the uptake of exogenous ARGs. This included nonsteroidal anti-inflammatories, ibuprofen, naproxen, diclofenac, the lipid-lowering drug, gemfibrozil, and the β-blocker propranolol. Based on the results of flow cytometry, whole-genome RNA sequencing and proteomic analysis, the enhanced transformation of ARGs was affiliated with promoted bacterial competence, enhanced stress levels, over-produced reactive oxygen species and increased cell membrane permeability. In addition, a mathematical model was proposed and calibrated to predict the dynamics of transformation during exposure to non-antibiotic pharmaceuticals. Given the high consumption of non-antibiotic pharmaceuticals, these findings reveal new concerns regarding antibiotic resistance dissemination exacerbated by non-antibiotic pharmaceuticals.

## Introduction

Antibiotic resistance is not a new phenomenon, but the recent increasing prevalence of antibiotic resistant bacteria and antibiotic resistance genes (ARGs) is unprecedented [1, 2]. Drug-resistant strains of bacteria first appeared in hospitals where most antibiotics are being used [3]. However, they are now frequently detected in the wider environments of water, soil and air [4–6]. It is evident that antibiotic resistance can spread among various environments, and ARGs are being exchanged between environmental bacteria and clinical pathogens.

The exchange process is mediated by horizontal gene transfer (HGT), including conjugation, transformation and transduction. Uniquely among them, transformation is only determined by the genes located on the recipient bacterial chromosome [7, 8]. Transformation is the direct uptake and incorporation of exogenous genetic elements, cell-free DNA such as plasmids, from the surroundings of the bacterium. This free DNA can enter the cell through its membranes, and may be expressed as a functional part of the bacterium [9]. In order to accomplish transformation, the bacteria need to be competent. More than 80 species of bacteria, both gram-positive and gram-negative, are reported to be naturally competent in the environment [8]. Due to the release of DNA from dead or damaged bacteria, cell-free DNA is abundant and ubiquitous in most environments. It is seen to persist for timespans of several hours to several months, depending on the surrounding environmental conditions. For example, free DNA can be stabilised by binding to mineral surfaces or humic substances [10]. Cell-free DNA possess a high possibility of encoding ARGs, as these are frequently located on plasmid DNA [7, 11, 12]. The plasmid-borne exogenous ARGs are found to be highly persistent in various environments and this may be due to the protection by organic matter [13]. Therefore, considering the ubiquitous occurrence and high abundance of cell-free DNA encoding ARGs in the environment, and the wide distribution of competent bacteria, transformation is an important pathway for ARG dissemination [11, 14].

Antibiotics are recognised as the most important drivers for accelerating transformation of ARGs. Antibiotics will impose selective pressure on bacteria, thus driving the spread of existing and newly arising ARGs [15]. As well, antibiotics can act as external stimuli to induce or enhance bacterial competence [16, 17]. Antibiotics can also facilitate bacterial lysis. This could result in release of increased levels of chromosomal or plasmid DNA that may harbour ARGs into the environment [18, 19]. The roles of antibiotics on transformation of ARGs are well studied [20]. However, although non-antibiotic pharmaceuticals make up more than 95% of the drug market worldwide [21, 22], their effect on DNA transformation is largely unknown. Recently, it was found that more than 200 non-antibiotic pharmaceuticals imposed antibiotic-like effects on human gut bacteria [23]. Our recent study also reports that carbamazepine (an anticonvulsant drug) promoted gene transfer through conjugation [24]. Consequently, it is of high interest to determine whether these non-antibiotic pharmaceuticals promote HGT of ARGs via the transformation process.

To fill this knowledge gap, we explored the potential of commonly consumed non-antibiotic pharmaceuticals to enhance the transformation of plasmid-borne ARGs. Six pharmaceuticals belonging to four categories were tested. This included three nonsteroidal anti-inflammatory drugs (NSAID), ibuprofen, naproxen and diclofenac, a lipid-lowering drug, gemfibrozil, the β-blocker, propranolol and a contrast medium, iopromide. All these pharmaceuticals are on the World Health Organization’s List of Essential Medicines and have large global consumptions. For example, for the NSAID alone, 30 million people worldwide are consuming these daily [25]. In addition, it is of concern that 90% of the pharmaceuticals consumed by humans are excreted and end up in wastewater [26, 27]. These are now considered to be emerging environmental contaminants. These drugs are frequently being detected in various environmental settings, that includes in wastewater, groundwater, surface water and drinking water, at concentrations ranging from nanograms to milligrams per litre [28, 29].

In this study, we established a transformation model of exogenous ARGs by exposing the naturally competent bacterium *Acinetobacter baylyi* (*A. baylyi*), to the free plasmid pWH1266, that encodes resistance towards ampicillin and tetracycline. We selected six non-antibiotic pharmaceuticals to test if they could significantly promote transformation frequency of ARGs at clinically and environmentally relevant concentrations. The underlying mechanisms facilitating the increased transformation were revealed by phenotypic and genotypic analyses, which included a culture-based transformation assay, measurement of reactive oxygen species (ROS) and cell membrane permeability, whole-genome RNA sequencing and proteomic analysis. Moreover, we developed a mathematical model to describe the extent of transformation during the long-term exposure of bacteria to non-antibiotic pharmaceuticals. Our findings provide strong evidence that non-antibiotic pharmaceuticals are playing considerable roles in the dissemination of antibiotic resistance.

## Materials and methods

### Bacterial strain and cell-free plasmid

The naturally competent *A. baylyi* ADP1 [30, 31] was used in this study. The plasmid, pWH1266 (8.89 kb) which encodes resistance to ampicillin (*bla*_TEM-1_) and tetracycline (*tetA*), was extracted from *Escherichia coli* (*E. coli*) (ATCC® 77092™) by Invitrogen™ PureLink® Quick Plasmid Miniprep Kit (Life Technologies, USA), and used as the exogenous gene transfer agent [32].

### Determining transformation frequencies during exposure to non-antibiotic pharmaceuticals

*A. baylyi* ADP1 was grown overnight in LB broth at 30°C. Then, 50 μL of the overnight culture was used to inoculate 5 mL of fresh LB (1%, v/v) in a 50 mL Falcon tube. The tube was incubated horizontally at 30 °C for 6 h in a shaking incubator when the culture had reached early stationary growth phase [33]. This was measured as an OD_600_ of 1.1, which correlates to a bacterial concentration of 3 × 10^8^ cfu/mL (Fig. S3). The bacteria were then harvested by centrifugation. The bacterial pellet was washed twice in PBS, and finally re-suspended in PBS solution. The free pWH1266 plasmid was prepared and suspended in elution buffer (10 mM Tris-HCl). To establish the transformation system, the plasmid was added to the *A. baylyi* ADP1 culture at a final concentration of 0.8 ng/μL (which calculates to 8.34 × 10^7^ copies/μL). Sodium acetate at a final concentration 100 mg/L was added to the transformation system as an energy source. The transformation system was then distributed into 1 mL aliquots in Eppendorf tubes and different levels of the non-antibiotic pharmaceuticals were added to each of the aliquots. Clinically and environmentally relevant concentrations, and subinhibitory concentrations of the pharmaceuticals were used (Tables S8–S10). These concentrations were 0.005, 0.05, 0.5, 5 and 50 mg/L for ibuprofen, naproxen, gemfibrozil, diclofenac and propranolol, and 0.01, 0.1, 1, 5 and 50 mg/L for iopromide. All of the transformation conditions were conducted at the least in biological triplicates.

After 6-h incubation at 25 °C without shaking, the successful transformation happened upon *A. baylyi* uptaking free pWH1266 plasmid. Thus, transformants (*A. baylyi* harbouring pWH1266 plasmid, with resistance to ampicillin and tetracycline) were formed. To count the number of transformant, 50 μL of the mixture from the transformation systems was spread onto LB agar selection plates, which contained 100 mg/L ampicillin and 10 mg/L tetracycline. The initial wild-type bacteria *A. baylyi* ADP1 without free plasmid was incubated under the same conditions except in the absence of the added plasmid, and spread onto the selective plates, to ensure no growth of the un-modified bacteria occurred. The total number of bacteria was also estimated by spreading the transformation systems onto LB agar in the absence of added antibiotics. The transformation frequency for each transformation system was calculated as the number of transformants divided by the total number of bacteria. Transformation in non-antibiotic pharmaceutical-dosed groups was compared with the corresponding control groups.

The effects of ROS were tested on the transformation process. One of the most commonly applied ROS scavengers, thiourea, which can eliminate the generated ROS in bacteria [34], was added at 100 μM to additional transformation assays prepared as described above.

### Verification of transformant

Transformants growing on the selective plates were randomly picked and cultured. The minimum inhibition concentrations (MICs) of ampicillin and tetracycline to the transformants were determined as previously described [24]. Plasmids were extracted from transformants using the Invitrogen™ PureLink® Quick Plasmid Miniprep Kit (Life Technologies, USA). The two ARGs on the plasmids were verified by PCR are described in Text S1 and Table S1. The extracted plasmids and PCR products were visualised following electrophoresis on 1% agarose gels.

### Measurement of ROS and cell membrane permeability

Bacterial ROS generation and cell membrane permeability were measured after the exposure to non-antibiotic pharmaceuticals. The 2′,7′-dichlorofluorescein diacetate (DCFDA) dye was used to detect cellular ROS, while propidium iodide (PI) dye was used to measure enhanced membrane permeability [35]. A CytoFLEX S flow cytometer (Beckman Coulter, USA) was applied to detect the fluorescence. Details of these methods are described in Text S2.

### Whole-genome RNA sequencing analysis

Whole-genome RNA sequencing was conducted to reveal gene expression levels during the transformation process. The same transformation systems were established as described above. Bacteria respond quickly to external stress on the transcriptional level. Thus, based on previous studies an exposure time of 2 h to non-antibiotic pharmaceuticals was applied [36, 37]. Total RNA was extracted from the transformation systems after exposure to 5 mg/L of ibuprofen, naproxen, gemfibrozil, diclofenac, propranolol or iopromide. Extractions were done using the RNeasy Mini Kit (QIAGEN®, Germany) except the protocol included an extra bead-beating step for cell lysis [24]. Biological triplicates of the extracted RNA were submitted to Macrogen Co. (Seoul, Korea), where Illumina paired-end sequencing was performed (HiSeq 2500, Illumina Inc, San Diego, CA). Gene expression levels between the control (no added pharmaceuticals) and the pharmaceutical-exposed groups were compared. Significant differences were assigned when both the *P* value and false discovery rate (*q* value) were <0.05. Details of the bioinformatics pipeline are described in Text S3.

### Proteomic analysis and bioinformatics

Proteomic analysis was applied to determine the relative protein abundance levels during the transformation process. The same transformation systems were established as described above. An exposure time of 6 h to the pharmaceuticals was applied for the proteomic investigations. This enables enough time to detect changes in the levels of proteins expressed in response to an external stress [38]. Bacterial total protein was extracted after exposure to 5 mg/L of ibuprofen, naproxen, gemfibrozil, diclofenac, propranolol or iopromide. Label free sequential window acquisition of all theoretical mass spectra (SWATH-MS) was applied for relative quantitative protein determination on the triplicate biological samples [39]. A stringency cut-off of *q* < 0.01 was applied. Details of the protein extraction, peptide preparations and bioinformatic pipelines are described in Text S4.

### Transformation modelling and computer simulation

An ordinary differential equation (ODE) model was proposed to simulate and predict the transformation process. The model is based on the dynamics of wild-type bacteria and transformant populations and the free plasmid pool (based on ref. [40]), and is described by the following differential equations:

1 $$\left\{ \begin{array}{l}\frac{{dN_0}}{{dt}} = r_0N_0\left( {1 - \frac{{N_0 + N_1}}{K}} \right) - d_0N_0 - \mu {{{\rm{PN}}}}_0\\ \frac{{dN_1}}{{dt}} = r_1N_1\left( {1 - \frac{{N_0 + N_1}}{K}} \right) - d_1N_1 + \mu {{{\rm{PN}}}}_0\\ \frac{{dP}}{{dt}} = - \mu {{{\rm{PN}}}}_0 + d_1N_1\lambda - \theta P\end{array} \right..$$

The first and second equations describe the number of wild-type (*N*_0_) and transformed bacteria (*N*_1_), three terms correspond to bacterial growth, death and transformation, respectively. The third equation describes the dynamics of free plasmids (*P*), indicating the plasmid uptake by wild-type bacteria, plasmid release due to the death of transformed bacteria and plasmid decay. All variables and parameters of the model are described in Table 1.

**Table 1:** Variables and parameters used in ODE model and implicit calibration model.

Symbol	Description
Model variables	
*N*_0_	Number of wild-type *A. baylyi*
*N*_1_	Number of transformant, *A. baylyi* with pWH1266 plasmid
*P*	Number of free plasmid
*t*	Time (h)
Model parameters	
*r*_0_	Growth rate of *N*_0_, set as 0.2
*r*_1_	Growth rate of *N*_1_, set as 0.2
*d*_0_	Death rate of *N*_0_
*d*_1_	Death rate of *N*_1_
*K*	Carrying capacity of the environment, set as 10^9^
*μ*	Transformation frequency
*λ*	Copy number of plasmid in transformant
*θ*	Decay rate of free plasmid in environment
*α*	Weight factor of the error between simulation and observation values of *N*_0_
*β*	Weight factor of the error between simulation and observation values of *N*_1_
Calibration parameters	
*μ*_ref_	Reference value of transformation frequency, 10^−^^8^
*d*_ref_	Reference value of death rate, 0.0125
*K*_*μ*_	Scale factor of transformation frequency
*K*_*d*_	Scale factor of death rate
*K*_μ_*	Optimal scale factor of transformation frequency
*K*_d_*	Optimal scale factor of death rate
LB_*μ*_	Lower bound of *K*_*μ*_
UB_*μ*_	Upper bound of *K*_*μ*_
LB_*d*_	Lower bound of *K*_*d*_
UB_*d*_	Upper bound of *K*_*d*_

The dosage of non-antibiotic pharmaceuticals may affect both the transformation frequency and the death rate. Thus, an implicit calibration model with two decision variables (*μ* and *d*) was introduced, and the objective function is described as follows:

2 $$	\min \,\,\, f\left( {K_\mu \times \mu _{{{\rm{ref}}}},K_d \times d_{{{\rm{ref}}}}} \right) \\ 	 \quad= \alpha \times \left[ {N_{0,{{\rm{obs}}}}\left( 6 \right) - N_{0,{{\rm{sim}}}}\left( 6 \right)} \right]^2 + \beta \times \left[ {N_{1,{{\rm{obs}}}}\left( 6 \right) - N_{1,{{\rm{sim}}}}\left( 6 \right)} \right]^2,$$

where *N*_0,obs_(6) and *N*_1,obs_(6) are the observed numbers of wild-type and transformed bacteria after 6 h, and *N*_0,sim_(6) and *N*_1,sim_(6) are the corresponding simulated values. The objective function is to minimise the square sum of errors between the observed numbers of both wild type and transformant, and their simulated values from the ODE model with specific parameters.

Explicit constraints of the two decision variables were:

3 $$\left\{ \begin{array}{l}{{{\rm{LB}}}}_\mu \le K_\mu \le {{{\rm{UB}}}}_\mu \\ {{{\rm{LB}}}}_d \le K_d \le {{{\rm{UB}}}}_d\end{array} \right..$$

Implicit calibration can be regarded as a closed circle containing two independent modules (optimisation tool and ODE simulation model, Fig. S1). Details are described in Text S5. The parameters and standard values used in the ODE model and the implicit calibration model are shown in Table 1.

Under the exposure of various non-antibiotic pharmaceuticals (5 mg/L for each pharmaceutical), the corresponding measured *N*_0,obs_(6) and *N*_1,obs_(6) were applied. The global optimal combinations of the two uncertain parameters (*K*_*μ*_ and *K*_*d*_) were calculated based on the ode15s function and the genetic algorithm solver in the optimisation toolbox 7.3 of MATLAB 2016b [41, 42] (details are described in Text S5, Figs. S1 and S2 and Tables S2–S7). To predict the combined effect of the six non-antibiotic pharmaceuticals, the combined amplify factor of *K*_*μ*_ (or *K*_*d*_) was assumed to be the product of the individual amplify factors in each pharmaceutical-dosage group. In addition, as all of the bacteria would become transformant (*N*_1_) in the long term, the stability time, defined as the time for reaching 95% of the final value, was calculated under different pharmaceutical-dosage conditions.

### Statistical analysis

Data were expressed as the mean ± standard deviation on figures. SPSS for Mac version 25.0 was applied for data analysis. Independent-sample *t*-tests were performed. As multiple comparisons were conducted for each pharmaceutical, the Bonferroni correction method was used to avoid spurious positives by multiplying *P* values with the number of comparison groups [43, 44]. The final calculated *P* values were shown as P^*^, and P^*^ values < 0.05 were considered to be statistically significant. All experiments were conducted in biological triplicates at least.

## Results

### Non-antibiotic pharmaceuticals increased the transformation frequency

**Fig. 1:**
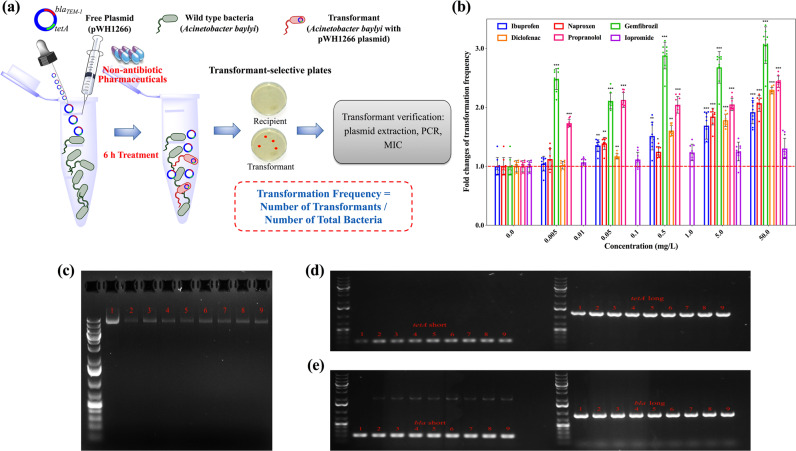
Transformation of free plasmid harbouring ARGs induced by non-antibiotic pharmaceuticals. **a** Schematic of the experimental design. **b** Fold changes of transformation frequency under the exposure of various concentrations of non-antibiotic pharmaceuticals, relative to pharmaceutical-free solvents. **c** Electrophoresis of plasmid pWH1266 (lane 1 is the original plasmid, lanes 2–3 are plasmids extracted from transformants of control with water and ethanol as the solvent, and lanes 4–9 are plasmids extracted from transformants of the pharmaceutical-dosed groups). **d** Electrophoresis of plasmid PCR products for *tetA* gene (lane 1 is the original plasmid, lanes 2–3 are plasmids extracted from transformants of control with water and ethanol as solvent, and lanes 4–9 are plasmids extracted from transformants of the pharmaceutical-dosed groups). **e** Electrophoresis of PCR products generated for the *bla* gene from the extracted plasmids (lane 1 is from the original plasmid, lanes 2–3 are from plasmids extracted from transformants of controls with water and ethanol as solvent, and lanes 4–9 are from plasmids extracted from transformants of the pharmaceutical-exposed groups). Significant differences between non-antibiotic-dosed samples and the control were analysed by independent-sample *t*-test and corrected by Bonferroni correction method, *P^*^ < 0.05, **P* < 0.01, and ***P* < 0.001.

The transformation systems were established by applying free plasmid pWH1266, encoding resistance against tetracycline and ampicillin, to the bacterial inoculum *A. baylyi*. The transformant *A. baylyi* with pWH1266 plasmid were formed upon successful transformation, and were quantified by transformant-selective plates. The transformation frequency was normalised as the number of transformants, after the 6-h exposure, to the total number of bacteria. During the transformation process, six kinds of commonly consumed non-antibiotic pharmaceuticals were tested (Fig. 1a).

In presence of ibuprofen, naproxen, gemfibrozil, diclofenac and propranolol, the absolute number of transformants increased and correspondingly, the transformation frequency also increased (Fig. S4, Table S11). For gemfibrozil and propranolol, the transformation frequencies began to increase significantly with dosage as low as 0.005 mg/L, which are clinically and environmentally relevant concentrations [45–47]. These frequencies further increased to 5.97 × 10^−6^ and 4.45 × 10^−6^ per total bacteria with dosages of 50 mg/L gemfibrozil and propranolol, respectively (P* = 0.00000002–0.00000008). Ibuprofen at concentrations higher than 0.05 mg/L (clinically and environmentally relevant) [29, 48], diclofenac with concentrations higher than 0.5 mg/L (clinically relevant) [49] and naproxen with concentrations higher than 5 mg/L enhanced the transformation frequency significantly (P* = 0.00000001–0.005) and reached 3.80 × 10^−6^, 4.16 × 10^−^^6^ and 4.07 × 10^−6^ per total bacteria, respectively, during exposure to 50 mg/L. In contrast, iopromide did not increase the transformation frequency at any exposure level, even at the high concentration of 50 mg/L (P* = 0.05-0.65). The fold changes of transformation frequency were determined by normalising the frequency with the corresponding solvent group of pharmaceuticals. Except for iopromide, all other non-antibiotic pharmaceuticals enhanced the transformation frequency, and the fold changes were 1.9-, 2.1-, 3.0-, 2.3-, 2.4-fold, at the exposure of 50 mg/L for ibuprofen, naproxen, gemfibrozil, diclofenac and propranolol, respectively (Fig. 1b). It is worth noting that the enhanced transformation frequency was due to the increased number of transformants, rather than the decreased number of total bacteria. The initial total bacterial concentration was 3 × 10^8^ cfu/mL, and during the incubating period of 6 h, the total number of bacteria did not change significantly compared with the control group (Table S11). This ruling out the contribution of cell death or vertical gene transfer to the increased transformation frequency.

Transformant cells from the various treated groups were randomly picked from the selective plates, and the transformation of the plasmid was verified by MIC measurement, plasmid extraction and PCR of the two specific ARGs on the plasmid, i.e., *tetA* and *bla* genes. While the wild-type *A. baylyi* was sensitive to tetracycline and ampicillin, the measured MIC indicated the randomly selected transformants had acquired resistance against tetracycline and ampicillin (Table S12). By electrophoresis it was seen that the plasmids from the randomly selected transformants were the same size as the original pWH1266 plasmid, and the specific PCR determined that the extracted plasmids harboured both *tetA* and *bla* genes (Fig. 1c–e). These verifications confirmed that the cell-free plasmid was successfully transformed into *A. baylyi*.

Collectively, five of the six selected non-antibiotic pharmaceuticals, including ibuprofen, naproxen, gemfibrozil, diclofenac and propranolol, enhanced the transformation frequency of ARGs at environmentally and clinically relevant concentrations. In contrast, iopromide did not affect the transformation frequency.

### Non-antibiotic pharmaceuticals increase ROS generation and affect the cell membrane

Under the effect of external stressors, this may cause bacteria to produce more ROS, stress responses may be triggered, and cell membranes may change [50, 51]. We hypothesised that these pharmaceuticals may cause stress to the bacteria and responses to the stress might enhance the DNA uptake and recombination, and thus promote the transformation effect. To test these hypotheses, ROS production and cell membrane permeability were measured by fluorescence approaches under the exposure of various concentrations of non-antibiotic pharmaceuticals.

**Fig. 2:**
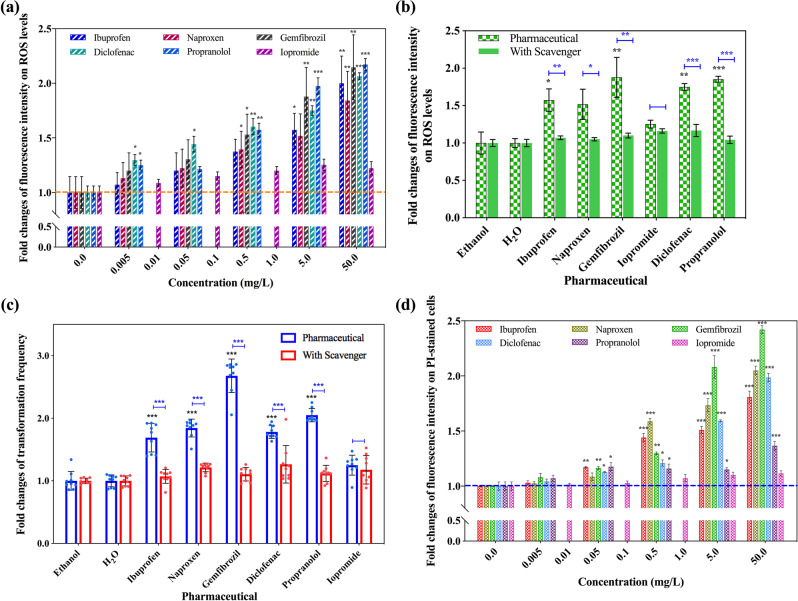
The effect of non-antibiotic pharmaceuticals causing increased ROS generation and altering the cell membrane integrity in *A. baylyi*. **a** Fold changes of ROS fluorescence intensity under the exposure of non-antibiotic pharmaceuticals. **b** Fold changes of ROS fluorescence intensity with the addition of the ROS scavenger thiourea (5 mg/L of non-antibiotic pharmaceuticals dosage). **c** Fold changes of transformation frequency with the addition of ROS scavenger thiourea and 5 mg/L of non-antibiotic pharmaceuticals. **d** Fold changes of fluorescence intensity on PI-stained cells under the exposure of non-antibiotic pharmaceuticals. Significant differences between non-antibiotic-dosed samples and the control were analysed by independent-sample *t*-test and corrected by Bonferroni correction method, *P* < 0.05, **P* < 0.01 and ***P* < 0.001. For figures **b** and **c**, significant differences between the groups with and without thiourea dosage were analysed by independent-sample *t-*test, *P < 0.05, **P < 0.01 and ***P < 0.001.

ROS levels were measured by application of the DCFDA dye and flow cytometry, during which the generation of hydrogen peroxide (H_2_O_2_), hydroxyl radicals (•OH), peroxynitrite (ONOO^−^) were detected. To examine for the effects on ROS generation, the non-antibiotic pharmaceuticals were applied separately at the same concentrations as in the transformation experiments and compared with the corresponding control group. It was seen that these pharmaceuticals at concentrations between 0.5 and 50 mg/L enhanced the ROS generation (Fig. S5). The increases in fold changes were enhanced with increasing concentrations of all pharmaceuticals except iopromide (Fig. 2a). The exposure to the five pharmaceuticals at 50 mg/L caused the greatest increases of the ROS generation, the fold changes were 2.0-, 1.8-, 2.2-, 2.1-, 2.2-fold (P* = 0.00001–0.009). In comparison, exposure to 50 mg/L of iopromide enhanced ROS generation by only 1.2-fold (P* = 0.060). These differences in ROS generation between iopromide and the other pharmaceuticals may partially explain why iopromide did not increase the transformation frequency. Notably, the DCFDA-positive fluorescence was due to the increase of bacterial ROS generation, there was no pharmaceutical-produced fluorescence in the assay.

ROS can be eliminated by scavengers, and thiourea is one of the most commonly applied scavengers [34]. Transformation systems with pharmaceutical exposure were established with the addition of 100 μM thiourea. Across all concentrations of all the pharmaceuticals, ROS production decreased to the levels detected in the controls. For the control groups, the addition of thiourea had no effect on the ROS generation, indicating the decrease of ROS generation in pharmaceutical-dosage groups was due to the elimination of radicals, instead of the dampen effects caused by the thiourea addition (Fig. S5). Significant decreases were also observed when comparing ROS generation between the exposure of the same concentration of pharmaceutical with and without thiourea (except for iopromide) (*P* = 0.0000002–0.032). For example, during exposure to 5 mg/L of ibuprofen, naproxen, gemfibrozil, diclofenac and propranolol, thiourea decreased the fold changes of ROS production from 1.6- to 1.1-, 1.5- to 1.1-, 1.9- to 1.1-, 1.8- to 1.2- and 2.0- to 1.1-fold, respectively. In contrast, adding thiourea to the 5 mg/L iopromide treatment did not decrease ROS production significantly (*P* = 0.06), with the fold change decreased from 1.3 to 1.2 (Fig. 2b). Again, these results are in accordance with the transformation changes affected by these non-antibiotic pharmaceuticals.

Based on these results, we would expect that the effect of ROS can also be reversed by adding thiourea during the transformation process. Indeed, it was observed that thiourea eliminated the enhanced effects of the non-antibiotic pharmaceuticals on transformation (Fig. S6). For example, under the exposure of 5 mg/L ibuprofen, naproxen, gemfibrozil, diclofenac, and propranolol, the presence of 100 μM thiourea decreased the transformation frequency significantly in comparison with the no thiourea systems (*P* = 0.000001–0.003) (Fig. 2c). Overall, neither the transformation frequency nor the fold change of frequency was significantly different in presence of both thiourea and the non-antibiotic pharmaceuticals from the corresponding control group (P* = 0.29–0.92). Collectively, these phenotypic results of ROS production, ROS production with the scavenger and the observed transformations correspond well, and indicate that the increased production of ROS imposed by non-antibiotic pharmaceuticals had an influence to cause the enhanced transformation of ARGs.

Another influence on the plasmid transformation would be the cell membrane permeability [52–54]. Thus, we measured changes in cell membrane permeability using the dye PI and flow cytometry. During exposure of bacterial cells to the pharmaceuticals, we detected increased PI-based fluorescence in comparison with the non-exposure group. Thus, it indicated the non-antibiotic pharmaceuticals caused increased cell membrane permeability (Fig. S7). It is worth to note that there was no change in fluorescence when the non-antibiotic pharmaceuticals alone were stained with PI. The detected differences in permeability were normalised to the corresponding control group as fold changes (Fig. 2d). Generally, the fold change of cell membrane permeability was enhanced with the increasing concentrations of pharmaceuticals, and peaked at the 50 mg/L of pharmaceutical dosage. These fold changes were 1.8-, 2.1-, 2.4-, 2.0- and 1.4-fold (P* = 0.000005–0.0016) for ibuprofen, naproxen, gemfibrozil, diclofenac and propranolol, respectively. In contrast, iopromide, which did not promote transformation significantly, only increased the membrane permeability by a maximum 1.1-fold change at the concentration of 50 mg/L (P* = 0.055).

These phenotypic tests of ROS generation, in the presence and absence of scavenger, and the cell membrane permeability, provide explanations for how the non-antibiotic pharmaceuticals enhanced the transformation of the cell-free ARGs.

### Non-antibiotic pharmaceuticals enhance stress levels and competence ability

**Fig. 3:**
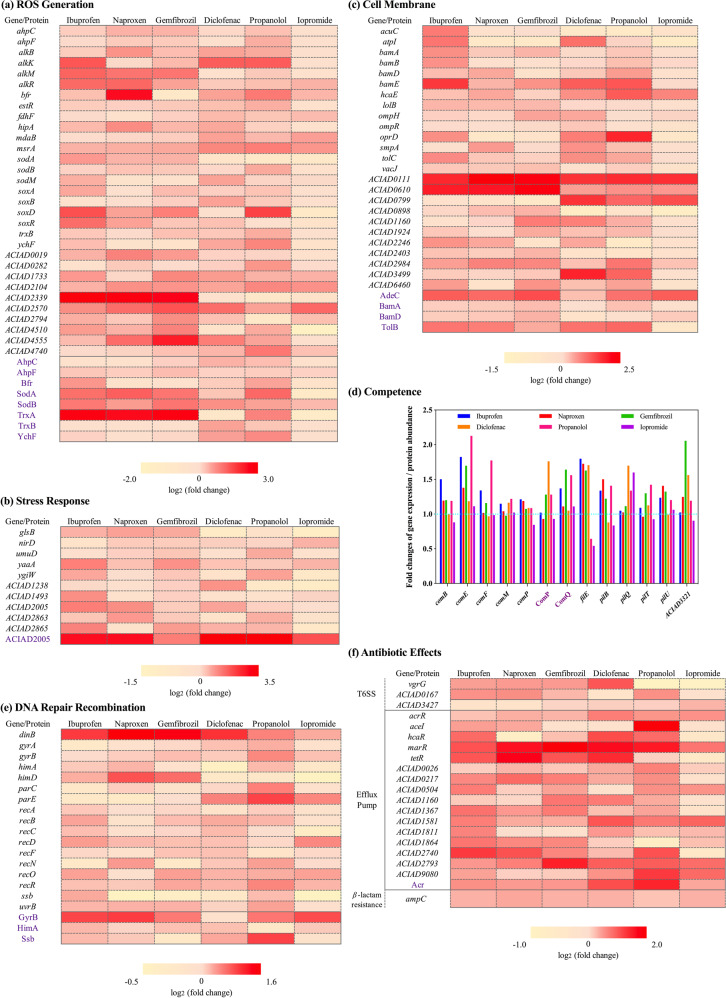
Genotypic RNA and protein analyses for *A. baylyi* under the exposure of non-antibiotic pharmaceuticals. **a** Log_2_ fold changes of key genes and proteins related to ROS generation. **b** Log_2_ fold changes of key genes and proteins related to stress response. **c** Log_2_ fold changes of key genes and proteins related to cell membrane status. **d** Fold changes of key genes and proteins related to bacterial competence. **e** Log_2_ fold changes of key genes and proteins related to DNA repair and recombination. **f** Log_2_ fold changes of key genes and proteins related to antibiotic effects.

In order to further explore the underlying mechanisms of the pharmaceutical enhanced transformation, the expression levels of genes and the abundances of proteins between the non-antibiotic pharmaceutical-dosed groups and the control group were compared. This was done by genome-wide RNA sequencing and measuring protein abundances. In accordance with the phenotypic effects, genes and proteins related to ROS and cell membrane showed significant upregulation upon bacterial exposure to non-antibiotic pharmaceuticals. In particular, genes coding for alkyl hydroperoxide reductase (*ahpCF*) [55], alpha-ketoglutarate-dependent dioxygenase (*alkBKMR*) [56, 57], hydrogen peroxide-inducible genes activator (*estR*) [58] and superoxide dismutase (*sodABM*) [55], all exhibited upregulation under the effects of 5 mg/L ibuprofen, naproxen, gemfibrozil, diclofenac and propranolol, while no significant enhancement was seen with 5 mg/L iopromide (Fig. 3a, Table S13). For example, gene *alkK* showed an enhanced regulation of 2.9-, 1.1-, 1.3-, 2.7-, 2.8-fold under the exposure of 5 mg/L ibuprofen, naproxen, gemfibrozil, diclofenac, propranolol, respectively. In contrast, iopromide had no effect on *alkK* expression compared with the control. Similarly, exposure to the non-antibiotic pharmaceuticals (except iopromide) significantly upregulated the expression levels of *ahpC*, *alkBR*, *estR*, *hipA*, *sodB* and *trxB*. The redox-sensing gene *soxR*, is a regulator responding to oxidative stress [55], also showed an enhanced expression under the exposure of 5 mg/L ibuprofen, naproxen, gemfibrozil and diclofenac, with 2.4-, 1.6-, 1.2- and 1.2-fold increases detected, respectively; while in the presence of iopromide a 0.68-fold change occurred compared with the control.

Corresponding to the changes detected in RNA expression, the abundance of ROS-related proteins changed significantly under the exposure of these non-antibiotic pharmaceuticals (*q* < 0.01). For example, the abundances of AhpC and AhpF were seen to vary, with the fold changes ranging from 0.85 to 1.4, in which iopromide had the least effect on the protein abundance (Fig. 3a, Table S14). Abundances of SodA and SodB also showed variations. Apart from iopromide, the other five non-antibiotic pharmaceuticals caused increased abundance of SodA and SodB. These increases ranged from 1.2- to 2.7-fold, while iopromide caused abundance changes of 0.5- to 1.3-fold. Moreover, in conjunction with the enhanced oxidative stress detected under pharmaceutical exposure, stimulation of universal stress and stress responses of *A. baylyi* were detected from significant upregulation of relevant proteins and genes (Tables S15, S16). For example, the abundance of universal stress protein ACIAD2005 was significantly upregulated under exposure to the non-antibiotic pharmaceuticals. The protein abundance was increased by 5.4-, 5.4-, 2.1-, 9.2- and 10.5-fold when exposed to 5 mg/L of ibuprofen, naproxen, gemfibrozil, diclofenac and propranolol, respectively. The corresponding genes were also enhanced, the increases ranging from 1.1- to 2.1-fold (Fig. 3b). Other genes related to stress response (*umuD*, *glsB*, *nirD*, yaaA and *ygiW*) in *A. baylyi* were also upregulated [59–61], the highest was a 2.0-fold increase under the exposure of 5 mg/L ibuprofen. Notably, of the 11 genes related to universal stress and stress response, exposure to 5 mg/L iopromide only caused minor increases in expression levels of three genes (1.1- to 1.4- fold), while the other five pharmaceuticals enhanced expression levels of at least six genes. This indicates that compared with the other pharmaceuticals, iopromide affected the least of a stress response in *A. baylyi*. These genotypic results on stress-related proteins and genes also contribute to explain why these five non-antibiotic pharmaceuticals enhanced the transformation of free ARGs, while iopromide did not.

Regarding genes and proteins related to cell membrane, upregulation were also seen with the dosage of non-antibiotic pharmaceuticals (Tables S17 and S18). Outer membrane protein assembly factors BamA and BamD for *Acinetobacter* increased significantly [62] (*q* < 0.01), with up to 1.3-fold increase detected (Fig. 3c). The TolB protein, part of the Tol-Pal system in *Acinetobacter*, plays a role in outer membrane invagination and is important for maintaining outer membrane integrity [63]. This protein showed significantly enhanced abundance under the exposure of 5 mg/L ibuprofen, naproxen, gemfibrozil, diclofenac and propranolol, these abundance changes ranging from 1.3- to 1.9-fold increases. Conversely, the abundance of TolB decreased by 0.7-fold under the effect of 5 mg/L iopromide. In addition, the membrane-related genes, *bamABDE*, *lolB*, *ompHR*, *oprD* and *tolC*, were upregulated during the exposure to the non-antibiotic pharmaceuticals (Fig. 3c). For example, a 3.0-fold increased expression of *oprD* was seen with exposure of 5 mg/L propranolol (*P* = 0.00005, *q* = 0.0006). Expression levels of *bamE* increased by 1.3- to 2.6-fold under the effect of ibuprofen, naproxen, gemfibrozil, diclofenac and propranolol, while iopromide had no effect. Exposure to iopromide did not cause increased expression levels of *bamAD*, *lolB*, *ompR*, *oprD*, *smpA* and *tolC* when compared with the control group. These limited effects of iopromide on the cell membrane on molecular levels, also explained why iopromide had less effect on transformation of the free plasmid compared with other five non-antibiotic pharmaceuticals.

As *A. baylyi* is naturally competent, in addition to the regulation of ROS, stress- and cell membrane-related genes, the presence of non-antibiotic pharmaceuticals also caused changes in the expression levels of proteins and genes related to natural transformation of *A. baylyi*. Type IV pilus regulators are associated with competence in *A. baylyi*, and these include the Com, Pil and Fil families [30, 64–66]. In this study, abundances of ComP and ComQ increased significantly under the exposure of non-antibiotic pharmaceuticals (*q* < 0.01). For example, exposure to 5 mg/L diclofenac increased abundance of ComP by 1.8-fold, and 5 mg/L gemfibrozil enhanced abundance of ComQ 1.6-fold (Fig. 3d). Compared with the other five non-antibiotic pharmaceuticals, iopromide had the least effect on abundance of both ComP and ComQ, with only 0.9- and 1.1-fold variations detected, respectively. For the mRNA sequencing, genes related to natural transformation were detected with altered expression, such as the genes *comBEFMP*, *filE*, *pilBQTU*. For example, *comE*, one of the most important genes for natural transformation of *A. baylyi* [65], was upregulated significantly under the exposure of all six non-antibiotic pharmaceuticals with changes of 1.8-, 1.4-, 1.7-, 1.2-, 2.1-, and 1.1-fold detected for ibuprofen, naproxen, gemfibrozil, diclofenac, propranolol and iopromide, respectively. Unlike the other non-antibiotic pharmaceuticals applied, downregulation of the genes *comBFP*, *filE* and *pilBT* were seen with 5 mg/L iopromide. The effects on genes involved in natural competence of *A. baylyi* imposed by the non-antibiotic pharmaceuticals also help to explain why these pharmaceuticals enhanced transformation efficiency.

In addition, expression levels of genes and proteins related to DNA repair and recombination also varied under the exposure of these non-antibiotic pharmaceuticals [59, 67, 68]. For example, the proteins GyrB, HimA and Ssb had significantly enhanced abundance (*q* < 0.01). The average fold changes of GyrB abundances were 1.8, 1.9, 1.5, 1.1, 1.5 and 1.8 during exposure to 5 mg/L ibuprofen, naproxen, gemfibrozil, diclofenac, propranolol and iopromide, respectively (Fig. 3e, Table S19). Also, a 1.2-fold increase of HimA was caused under the exposure of 5 mg/L ibuprofen, and a 1.9-fold change of Ssb was shown with the effect of 5 mg/L propranolol. Compared with these changes in protein abundance, more changes in genes expression relating to recombination were detected (Table S20). For example, the gene *recD*, which is reported to affect plasmid maintenance and recombination [67], was upregulated when exposed to these non-antibiotic pharmaceuticals by 1.1- to 1.4-fold. The expression of gene *dinB*, reported to be induced by DNA damage [59], showed significant upregulation by up to 2.9-fold. Notably, among the 17 genes related to DNA damage and recombination, only 8 genes showed upregulation under the exposure of iopromide, while the expression levels of 10–15 genes increased with the dosage of the other five non-antibiotic pharmaceuticals.

Interestingly, these genotypic investigations also revealed that under the exposure of these non-antibiotic pharmaceuticals, *A. baylyi* showed responses that are related to antibiotic exposure type responses. A core regulator of the antimicrobial resistance-related Type VI secretion system (T6SS) in *Acinetobacter*, the VgrG family, is a potent mediator of antibacterial activity during interbacterial interactions [69, 70]. The expression level of gene *vgrG* was enhanced under the exposure of non-antibiotic pharmaceuticals (except for propranolol and iopromide) by 1.4- to 2.0-fold (Fig. 3f, Table S21). In addition, the non-antibiotic pharmaceuticals stimulated efflux pump genes. For example, genes belonging to the drug efflux regulatory Mar family [71] showed significant upregulations. The dosage of 5 mg/L ibuprofen, naproxen, gemfibrozil, diclofenac, propranolol and iopromide enhanced the expression of *marR* by 2.1-, 3.0-, 3.8-, 3.1-, 2.9- and 1.7-fold, respectively (Fig. 3f, Tables S22 and S23). The TetR/AcrR family also mediates multidrug efflux pumps in bacteria [71–73]. We detected the non-antibiotic pharmaceuticals increased the abundance of the Acr regulator significantly (*q* < 0.01), and also enhanced the expression of the corresponding *acr*, *tet* and *ace* genes. As high as 2.7-fold increased abundance of Acr was seen with 5 mg/L propranolol. Correspondingly, the expression levels of *acrR*, *tetR* and *aceI* increased by 1.4-, 1.1- and 3.9-fold, respectively (Fig. 3f). In addition, the non-antibiotic pharmaceuticals promoted the expression of *ampC* (Table S24), which is related to resistance towards β-lactam antibiotics [74].

We also found that the exposure of non-antibiotic pharmaceuticals caused large-scale changes in the expression of TonB-related proteins and genes. TonB of *Acinetobacter* is reported to play key roles in biofilm formation, cell adhesion and is closely associated with virulence [75, 76]. For all the 31 genes related to TonB detected, most of them (maximum 24 genes) showed upregulation under the exposure of the non-antibiotic pharmaceuticals (Tables S25 and S26). A possibility here is that the non-antibiotic pharmaceuticals stimulated the TonB activity to promote cell adhesion. As bacterial competence and biofilm development are mediated and regulated by many of the same gene products [77], we propose here that TonB-related upregulation also contributes to the enhanced transformation.

### Non-antibiotic pharmaceuticals impose accumulated effects on transformation

Due to the limitation of the time span (6 h) in our transformation experiments, an ODE mathematical model was proposed to simulate and predict the long-term effects of transformation (more than 500 h) under the exposure of non-antibiotic pharmaceuticals. In addition, as various pharmaceuticals often co-exist in both environmental and clinical settings [29, 47], we also studied the effect of exposure to multiple pharmaceuticals.

**Fig. 4:**
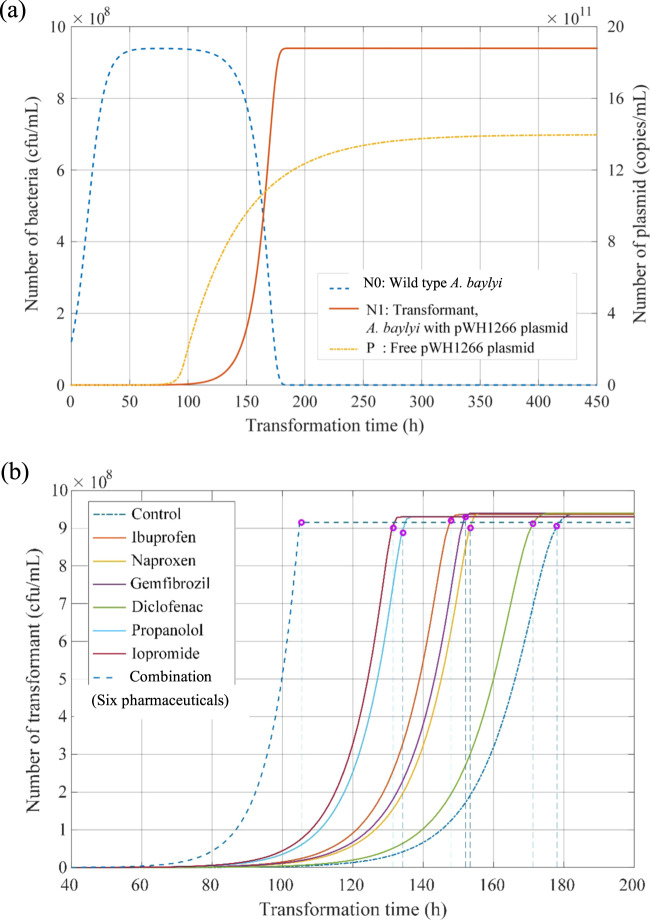
The simulated changes in the number of bacteria with the increase of transformation time. **a** Variation trends of wild-type bacteria, transformant and free plasmid in the control group. **b** The number of transformants and the corresponding stability time (shown as pink circles) under the exposure of different non-antibiotic pharmaceuticals.

The experimentally measured numbers of wild-type bacteria and transformants during exposure to 5 mg/L of pharmaceutical at the times of 0 h and 6 h were applied to calibrate the two uncertain parameters in the model. After model simulation and calculation, the trajectories of the numbers of wild-type bacteria (*N*_0_), transformant (*N*_1_) and free plasmid (*P*) in the long term were visualised (Fig. 4a). It was seen that the number of wild-type bacteria increased at first and then decreased to 0, while the number of transformants were 0 at the start point, and gradually increased to a steady value (9 × 10^8^ cfu/mL). Similar to previous findings [40, 78], all of the wild-type *A. baylyi* will eventually uptake free plasmid in the environment and become transformants after a sufficiently long time. Free plasmids will still be present because the death of transformants will continuously release them into the surrounding environment.

The optimal scale factors of transformation frequency (*K*_μ_*) and death rate (*K*_d_*) in the long term were quantitatively determined. The amplify factors of *K*_μ_* and *K*_d_* were correspondingly calculated by comparing with those in the control group (Table 2). It was predicted that long-term exposure to iopromide caused a 2.5-fold increase in transformation frequency, while the other five non-antibiotic pharmaceuticals imposed increases ranging from 5.5- to 19.0-fold. The fold changes were higher than those detected at the time point of 6 h, indicating the transformation effects imposed by non-antibiotic pharmaceuticals will accumulate with the increase of transformation time. It was predicted that the non-antibiotic pharmaceuticals imposed little effect on the long-term death rates, with the highest being a 1.3-fold change.

**Table 2:** Simulation and calculation results under the exposure of non-antibiotic pharmaceuticals.

Condition	*K*_μ_*	Amplify factor of *K*_μ_*	*μ*	*K*_d_*	Amplify factor of *K*_d_*	*d*	Stability time (h)^a^
Control	0.157	1	1.75 × 10^−9^	0.936	1	0.0117	178
Ibuprofen	0.856	5.5	9.54 × 10^−9^	1.109	1.1848	0.0139	148
Naproxen	1.230	7.8	1.37 × 10^−8^	0.946	1.0107	0.0118	153
Gemfibrozil	2.937	18.7	3.27 × 10^−8^	0.955	1.0203	0.0119	152
Diclofenac	1.576	10.0	1.76 × 10^−8^	1.161	1.2404	0.0145	134
Propranolol	2.984	19.0	3.33 × 10^−8^	1.193	1.2746	0.0149	131
Iopromide	0.395	2.5	4.41 × 10^−9^	0.969	1.0353	0.0121	171
Combined effect	–	–	–	Product	1.9998	0.0233	105

^a^The time reaching 95% of the final theoretical value.

As the number of transformants is predicted to remain at a high, stable level in the long term, we hypothesise that the exposure to non-antibiotic pharmaceuticals would affect the required time to reach the corresponding stable value. Thus, a “stability time” was proposed, which was defined as the time required to reach 95% of the final theoretical value. For the control group, 178 h was needed to reach the stable number of transformants. However, the pharmaceuticals decreased the stability time to 131 h under the exposure of propranolol (Fig. 4b and Table 2). In addition, the combination of all these pharmaceuticals accelerated the stability time to 105 h, which was 41.0% faster than that without any pharmaceutical dosage. Notably, the calculation of the stability time depends on both the simulated transformation frequency and the death rate. Thus, the combined effect on stability time accounted for both the combined effects on transformation frequency and death rate imposed by all six non-antibiotic pharmaceuticals. Collectively, the modelling of long-term transformation indicates that the non-antibiotic pharmaceuticals increase transformation frequency and accelerate the transformation process. As well, the combined effect of the six pharmaceuticals would further enhance the transformation.

## Discussion

The dissemination of antibiotic resistance is a serious concern to both human health and environmental ecosystems. Previous studies indicate that HGT is one of the most important pathways for dissemination of antibiotic resistance [79, 80]. Compared with conjugation and transduction, transformation is regarded as the only bacteria-encoded mode of gene transfer, as it is the process of direct uptake of exogenous DNA from the surroundings of a bacterium [7, 8]. Naturally transformable bacterial strains are widely detected in various environments, including soil, wastewater and surface water bodies [13, 81], and some clinically relevant bacterial strains also exhibit natural competence [82, 83]. In addition, plasmid DNA is one of the most detected cell-free exogenous DNA, having high persistence in natural environments, playing a key role in microbial evolution, and frequently encoding ARGs [13, 84, 85]. Therefore, the high prevalence of both transformable cells and free plasmid-borne ARGs enables the frequent occurrence of natural transformation, thus making it an important pathway for dissemination of antibiotic resistance that should not be neglected [11].

Non-antibiotic pharmaceuticals also occur in environments with elevated concentrations, ranging from nanograms to milligrams per litre (Table S8) [28, 29]. However, the relationship between non-antibiotic pharmaceuticals and the transformation of ARGs has been overlooked. In this study, we address this knowledge gap by establishing both experimental and mathematical transformation models, to mimic the natural transformation process. To achieve this, one of the most widely investigated species of *Acinetobacter*, *A. baylyi*, was chosen. It is naturally competent, frequently isolated from soil and activated sludge, and also reported as an opportunistic pathogen [86–88]. A small-sized plasmid, pWH1266 (8.89 kb), with tetracycline and ampicillin resistance was chosen as the free plasmid DNA [32].

Our phenotypic transformation experiments demonstrated that five of the six selected non-antibiotic human-targeted pharmaceuticals, that included the anti-inflammatory drugs (ibuprofen, naproxen and diclofenac), a lipid-lowering drug (gemfibrozil), a β-blocker (propranolol), when applied for 6 h at environmentally and clinically relevant concentrations (0.005–0.5 mg/L), could significantly enhance the transformation frequency of ARGs (P* < 0.05). This was also seen in the fold change of transformation frequency, which could be as high as three-fold. The fold changes imposed by non-antibiotic pharmaceuticals were seen to be within the same range in comparison to those induced by antibiotics. For example, ciprofloxacin is reported to increase transformation frequency by 3- to 4-fold in the gram-negative human pathogen *Helicobacter pylori* [17]. Exposure to nalidixic acid causes a 2- to 3-fold increase in transformation in *Legionella pneumophila* [89]. In contrast, a contrast medium (iopromide) used in this study (0.01–50 mg/L), did not seem to have an effect on the transformation process in the 6 h study.

We further investigated the pharmaceutical-promoted transformation process over longer time periods (more than 500 h) using a mathematical model. Excepting for iopromide (2.5-fold change), under the exposure of the other five non-antibiotic pharmaceuticals, 5.5- to 19.0-fold increased transformation frequencies were obtained. In comparison with the transformation experiments of 6 h, enhanced fold changes were seen with the increase of time, thus, indicating the promoted transformation can be accumulated. In addition to the transformation frequency, the non-antibiotic pharmaceuticals also accelerated the transformation process by as much as 26.4%. In other words, non-antibiotic pharmaceuticals facilitated the process of bacteria becoming transformable. Moreover, the combined effects were assessed as pharmaceuticals often co-exist in both environmental and clinical settings [29, 47]. The combination of all six pharmaceuticals could further magnify the enhanced transformation rate of cell-free ARGs by 41.0%.

**Fig. 5:**
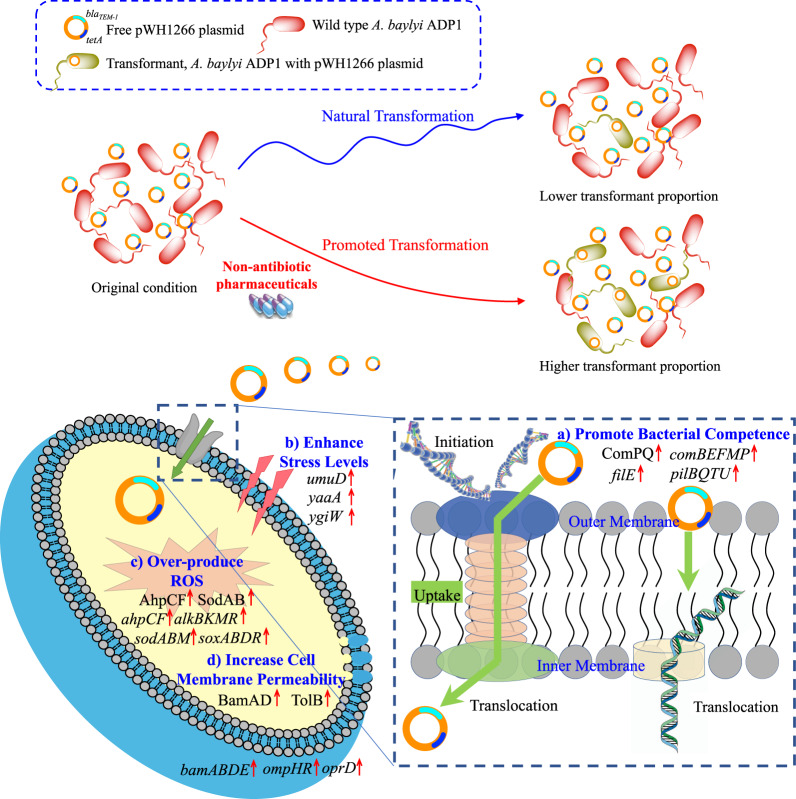
The overall mechanisms explaining the roles of non-antibiotic pharmaceuticals causing enhanced transformation of exogenous ARGs. **a** Non-antibiotic pharmaceuticals promote bacterial competence. **b** Non-antibiotic pharmaceuticals enhance stress levels. **c** Non-antibiotic pharmaceuticals induce over-production of ROS. **d** Non-antibiotic pharmaceuticals increase cell membrane permeability.

We also applied a series of molecular approaches to investigate the underlying mechanisms of the non-antibiotic pharmaceutical enhanced transformation of ARGs. By employing flow cytometry, as well as genome-wide RNA and protein sequencing, we propose that four factors are playing key roles in this enhanced transformation (Fig. 5). These include: (a) promoted bacterial competence, (b) enhanced stress levels, (c) over-produced ROS and (d) increased cell membrane permeability.

Bacterial competence is a physiological state, being regarded as the prerequisite for the transformation process; and competence development is one of the factors limiting transformation [90]. Some stressors, including starvation and limited water availability, can induce bacterial competence under artificial conditions [91, 92]. Exposure to antibiotics (e.g., β-lactams, aminoglycosides, fluoroquinolone and mitomycin C), UV radiation and water disinfection by-products can stimulate competence in many species of bacteria, including both naturally competent and non-competent bacteria [16, 17, 89, 93, 94]. However, no prior studies have shown that widely consumed non-antibiotic pharmaceuticals can stimulate competence. Here we show that under the effect of non-antibiotic pharmaceuticals, competence of *Acinetobacter* was promoted. This was evident from the significantly enhanced regulation of both competence regulons (*q* < 0.01), ComP and ComQ during exposure to the pharmaceuticals. Other competent-related proteins Pil, Fil, GyrB, HimA and Ssb families, and corresponding genes also showed increased expression levels [16, 67, 95]. In addition, our phenotypic investigations revealed the non-antibiotic pharmaceuticals caused increase of the proportion of transformants in the total bacterial community, instead of affecting bacterial growth. This further indicating that the bacterial physiological state had changed and bacterial competence was stimulated. Thus, we propose that the promoted competence ability of *Acinetobacter* caused by non-antibiotic pharmaceuticals is a main mechanism for enhanced transformation of ARGs.

Competence for genetic transformation is considered as the evolution of stress responses in bacteria species [16, 17, 95]. In this study, we noticed that universal stress and stress responses of *A. baylyi* were stimulated by the exposure to non-antibiotic pharmaceuticals. Enhanced stress levels can lead to the expression of DNA repair-recombination enzymes and promote free DNA uptake ability [96]. Our genotypic investigations revealed the increased expression levels of genes including *recA* and *umuD* [17, 97]. Thus, we imply that the promoted transformation of ARGs we observe here is linked to the enhanced stress levels caused by these non-antibiotic pharmaceuticals.

We also found ROS generation levels increased significantly with the dosage of the non-antibiotic pharmaceuticals. This increase was measured directly by fluorescence detection of ROS and supported by the changes detected in gene and protein expression. More than a twofold increase in ROS was seen under the effect of non-antibiotic pharmaceuticals (except for iopromide). This coincided with the significant upregulation of related genes, including *soxR*, *sodABM*, *ahpCF*, which are considered to be regulators in the oxidative stress response [55, 56]. The genotypic results relating to ROS corresponded with the phenotypic experiments, and indicate the increased levels of ROS contributed to the enhanced transformation effects. Interestingly, these non-antibiotic pharmaceuticals are also shown to increase ROS production in animal and human studies. For example, NSAID-pharmaceuticals are reported to induce cardiotoxicity by an ROS dependent mechanism [98]. Ibuprofen and naproxen cause increase in superoxide, hydroxyl radicals and further cause DNA strand scission [99, 100]. Naproxen increased ROS production in rat cardiac cells [98], and diclofenac was also reported to impose apoptotic effects on human and rat hepatocytes by increasing ROS levels, especially superoxide [101]. The lipid-lowering drug gemfibrozil is seen to enhance the ROS production in phagocytic leucocytes [102]. Importantly, in this study, we also reversed the effects of ROS generation on enhanced transformation by adding an ROS scavenger, thiourea. The inclusion of thiourea was seen to eliminate the promoted transformation effects caused by non-antibiotic pharmaceuticals completely. Therefore, increased production of ROS induced by the non-antibiotic pharmaceuticals is another important factor to influence the transformation of free ARGs.

Cell membranes will act as barriers for free plasmid entering the recipient bacteria [52, 103]. We detected that under the exposure of non-antibiotic pharmaceuticals, the cell membrane permeability was enhanced significantly as measured using the membrane integrity dye PI. Genome-wide sequencing indicated that protein abundance and gene expression related to cell membrane status were enhanced significantly with the addition of ibuprofen, naproxen, gemfibrozil, diclofenac and propranolol. The proteins included Bam, Omp and Tol families, which serve as membrane protein assembly factors and regulate cell membrane permeability [62, 63]. Other studies have detected similar genetic responses under the exposure of antibiotics. Exposure to colistin is seen to cause outer membrane damage, alter normal membrane composition in *Acinetobacter* and promote the uptake of free DNA [104]. Moreover, the most recognised competence-inducing stimuli, Ca^2+^ and Mg^2+^, are also illustrated to induce the formation of pore-like structures in the cell surface and thus facilitate the passing of double-stranded DNA, including circular plasmids [105]. Based on these findings, we speculate that the non-antibiotic pharmaceutical exposure altered the cell membrane and this plays an important role in the enhanced transformation of ARGs.

Interestingly, *A. baylyi* showed some other responses towards non-antibiotic pharmaceuticals that are similar to changes detected during exposure to antibiotics. For example, T6SS is a widespread secretory apparatus produced by gram-negative bacteria involved in antimicrobial resistance activity [69, 70]. This efflux pump has been well studied and is strongly implicated with bacterial antimicrobial resistance. The antibiotics colistin, doripenem and ciprofloxacin are seen to promote the efflux pump levels in *Acinetobacter* significantly [61, 104, 106]. In this study, we found that under the exposure of non-antibiotic pharmaceuticals, both antimicrobial resistance-related T6SS apparatus and efflux pump were stimulated, as detected from changes in the transcriptional and translational levels. Moreover, antibiotics are widely reported to accelerate evolution of virulence in transformable species [17, 63]. We also found that the virulence and cell adhesion regulon in *Acinetobacter*, the TonB protein abundances and corresponding gene expression levels were enhanced significantly under the exposure of non-antibiotic pharmaceuticals. Further studies are needed to investigate whether non-antibiotic pharmaceuticals could stimulate virulence of bacteria, and to investigate the details of the relationship between enhanced virulence and promoted dissemination of ARGs.

Collectively, this study offers evidence for the first time that commonly consumed non-antibiotic pharmaceuticals significantly promote the bacterial transformation of exogenous ARGs. Promoted bacterial competence, enhanced stress levels, over-produced ROS and increased cell membrane permeability are potentially contributing to the non-antibiotic pharmaceutical enhanced transformation of ARGs. Our study sheds new light on the spread of antibiotic resistance caused by non-antibiotic pharmaceuticals, it highlights the necessity to re-evaluate the antibiotic-like side effect of non-antibiotic pharmaceuticals, and broadens our view of antibiotic resistance. Further studies are required to test whether non-antibiotic pharmaceuticals can induce transformation in naturally non-competent bacteria, such as in *E. coli*. Also, these in vitro findings need to be tested rigorously in vivo (e.g., in animal models or clinical trials) for further exploration.

## Supplementary information

Supporting Information

## Data Availability

All data were deposited in publicly accessible databases. RNA sequence data are accessible through the Gene Expression Omnibus of NCBI (GSE142061). The mass spectrometry proteomics data have been deposited to the ProteomeXchange Consortium via the PRIDE [44] partner repository with the dataset identifier of PXD016798.
