# Correction: Non-antibiotic pharmaceuticals enhance the transmission of exogenous antibiotic resistance genes through bacterial transformation

**DOI:** 10.1038/s41396-021-01074-x

**Published:** 2021-08-02

**Authors:** Yue Wang, Ji Lu, Jan Engelstädter, Shuai Zhang, Pengbo Ding, Likai Mao, Zhiguo Yuan, Philip L. Bond, Jianhua Guo

**Affiliations:** 1 Advanced Water Management Centre, The University of Queensland, Brisbane, QLD 4072 Australia; 2 School of Biological Sciences, The University of Queensland, Brisbane, QLD 4072 Australia

**Keywords:** Antibiotics, Next-generation sequencing

Correction to: *The ISME Journal* 10.1038/s41396-020-0679-2

The article “Non-antibiotic pharmaceuticals enhance the transmission of exogenous antibiotic resistance genes through bacterial transformation”, written by Yue Wang, Ji Lu, Jan Engelstädter, Shuai Zhang, Pengbo Ding, Likai Mao, Zhiguo Yuan, Philip L. Bond and Jianhua Guo, was originally published Online First without Open Access. After publication in volume 14, issue 8, page 2179–2196 the author decided to opt for Open Choice and to make the article an Open Access publication. Therefore, the copyright of the article has been changed to © The Author(s) 2020 and the article is forthwith distributed under the terms of the Creative Commons Attribution 4.0 International License, which permits use, sharing, adaptation, distribution and reproduction in any medium or format, as long as you give appropriate credit to the original author(s) and the source, provide a link to the Creative Commons licence, and indicate if changes were made.

The images or other third party material in this article are included in the article’s Creative Commons licence, unless indicated otherwise in a credit line to the material. If material is not included in the article’s Creative Commons licence and your intended use is not permitted by statutory regulation or exceeds the permitted use, you will need to obtain permission directly from the copyright holder.

To view a copy of this licence, visit http://creativecommons.org/licenses/by/4.0/.

