# Supplementary material for: Non-antibiotic pharmaceuticals enhance the transmission of exogenous antibiotic resistance genes through bacterial transformation
Source: ISME J. 2020 May 18;14(8):2179–96. doi: 10.1038/s41396-020-0679-2 (PMC7367833; doi:10.1038/s41396-020-0679-2)
Supplement: Supplementary file 1 — Supporting Information [file 41396_2020_679_MOESM1_ESM.pdf]

Supporting Information

Non-antibiotic pharmaceuticals enhance the transmission of exogenous antibiotic resistance genes through bacterial transformation

Running title: Non-antibiotic drugs enhance uptake cell-free DNA

Yue Wang[1], Ji Lu[1], Jan Engelstädter[2], Shuai Zhang[1], Pengbo Ding[1], Likai Mao[1], Zhiguo Yuan[1], Philip L. Bond[1], Jianhua Guo[1,*].

[1] Advanced Water Management Centre, The University of Queensland, Brisbane, Queensland, Australia, 4072

[2] School of Biological Sciences, The University of Queensland, Brisbane, Queensland, Australia, 4072

* Corresponding author: j.guo@awmc.uq.edu.au

This file includes:

Supplementary Texts 1 to 5

Supplementary Figures 1 to 7

Supplementary Tables 1 to 26

Supplementary Texts

Text S1. PCR conditions

PCR systems were set up as 20 μL, with 10 μL Platinum™ Green Hot Start PCR Master Mix (2X) (Invitrogen™), 0.4 μL 20 μM primer, 1 μL plasmid, 2 μL GC solution, and 6.6 μL ddH_2O. Primers are listed in Supplementary table 1. PCR conditions for genes *tetA* and *bla* were: denaturation at 94 ^oC for 4 min on initial cycle, 30 s for another 35 cycles, annealing at 55 ^oC for 30 s, extension at 72 ^oC for 1 min, followed by 7 min. The process was conducted with 30 cycles [1].

Table S1. Primers used in this study [1,2]

Gene	Primer	Sequence of primer
tetA	Short FW	GACTATCGTCGCCGCACTTA
	Short RV	ATAATGGCCTGCTTCTCGCC
	Long FW	CGTGTATGAAATCTAACAATGCGCT
	Long RV	CCATTCAGGTCGAGGTGGC
bla	Short FW	AATAAACCAGCCAGCCGGAA
	Short RV	TTGATCGTTGGGAACCGGAG
	Long FW	TTACCAATGCTTAATCAGTGAGGC
	Long RV	ATGAGTATTCAACATTTCCGTGTCG

Text S2. ROS generation and cell membrane permeability detection

Bacterial culture of *Acinetobacter baylyi* ADP1 was washed twice with PBS and resuspended in PBS to reach 10^6 cfu/mL. For ROS detection, bacteria strains were incubated in dark at 37 ^oC for 30 min with 2', 7'-dichlorofluorescein diacetate (DCFDA, at a final concentration of 20 uM, abcam®). Then, 100 μL of the bacteria stained with DCFDA were treated with different concentrations of non-antibiotic pharmaceuticals. 1.5% H_2O_2 was set as positive control, and MilliQ water / ethanol was set as negative control. After complete mixing by vortex, the mixtures were incubated in dark at 25 ^oC for 2 h before measurement at 488 nm. For cell membrane permeability detection, 100 μL of bacteria strain was exposed to different concentrations of non-antibiotic pharmaceuticals, and incubated at 25 ^oC for 6 h. The same volume of MilliQ water / ethanol was the negative control, while bacteria strain treated with

100 °C water was the positive control. The strains were then stained with 1 μL of propidium iodide (PI, 2 mM, Life Technologies) and incubated in the dark for 15 min before measurement at 561 nm. All data was analysed with CytExpert. All the detections were conducted in triplicate. Relative fold increases in ROS production or cell membrane permeability were calculated as pharmaceutical-treated samples divided by the corresponding negative control samples (based on the solvent) according to previous studies [3,4].

Text S3. Whole-genome RNA sequence analysis and bioinformatics

After obtaining raw data from Macrogen Co. (Seoul, Korea), NGS QC Toolkit (v2.3.3), SeqAlto (version 0.5), and Cufflinks (version 2.2.1) were applied to treat the raw sequence reads and to analyse the differential expression for triplicated samples. The database used for alignment was the reference genome of *A. baylyi* ADP1 (NC_005966.1), obtaining from National Center for Biotechnology Information (NCBI). CummeRbund package in R was used to conduct the statistical analyses. The measure of "fragments per kilobase of a gene per million mapped reads" (FPKM) was applied to quantify gene expression. The differences of gene expression between the control (no added pharmaceuticals) and the pharmaceutical-exposed groups were presented as $\log_2$ fold-changes (LFC) [3,5]. Significant differences were seen when both *P* values and false discovery rate (*q* value) less than 0.05.

Text S4. Proteomics analysis

Total protein was extracted by B-PER™ Bacterial Protein Extraction Reagent. The extracted proteins were treated by reduction, alkylation, trypsin digestion, and ziptip clean-up procedures [6]. The peptide preparations were then loaded to mass spectrometer. Qualitative protein libraries were constructed by information dependent analysis (IDA); while quantitative protein determination was based on SWATH-MS using biological triplicate samples [6]. IDA data were combined and searched using ProteinPilot software, with the database of *Acinetobacter baylyi* (strain ATCC 33305 / BD413 / ADP1) (received from Uniprot on 12th of March 2019). Search setting for enzyme digestion was set to trypsin and alkylation was set to iodoacetamide. Afterwards, the constructed IDA library and SWATH-MS data were loaded into PeakView v2.1 for further processing, with the peptide confidence threshold of 99%, number of peptides per protein of 5, and number of transitions per peptide of 3. A minimum of 2 peptides and 3 transitions was used for quantitative analysis. A

stringency cut-off of q value less than 0.01 was used to identify the proteins with significant different expression levels compared with the control samples.

Text S5. Transformation modelling and computer simulation

Implicit calibration can be regarded as a closed circle containing two independent modules (optimization tool and ODE simulation model). The process of updating uncertain model parameters by applying an optimization tool (genetic algorithm, GA) is illustrated in Fig.S1. The initial population satisfying the corresponding constraints were first generated by a creation function and sent to the ODE simulation model. Afterwards, simulated values of N_0 and N_1 at time 6 h were calculated by an effective stiff solver (ode15s) in MATLAB 2016b. Generations specifies the maximum number of iterations the genetic algorithm performs. Based on these simulated values from ODE module, the initial population evolved between every two generations and finally reached a convergent value to the global optimization solution.

When applying the GA for model calibration with two decision variables, feasible subranges were introduced to find the global optimization point effectively [7,8]. Thus, smaller variation ranges of the two scale factors (K_μ and K_d) were put forward. Eight partitions of each decision variable were configurated based on the same benchmark points (composing set Φ, see Table S2). A-H represented the variation range of parameter K_μ, and a-h represented the variation range of parameter K_d (shown in Tables S3 and S4, respectively). Correspondingly, the feasible subranges of GA optimization can be regarded as the combination of variation ranges in Tables S3 and S4 (Table S5). Noticeably, the feasible subranges must enclose the benchmark point (L_μ, L_d) when setting constraints of GA, and the schematic diagram from point (L_1, L_2) to area Ω_μ-Ω_d is shown in Fig. S2.

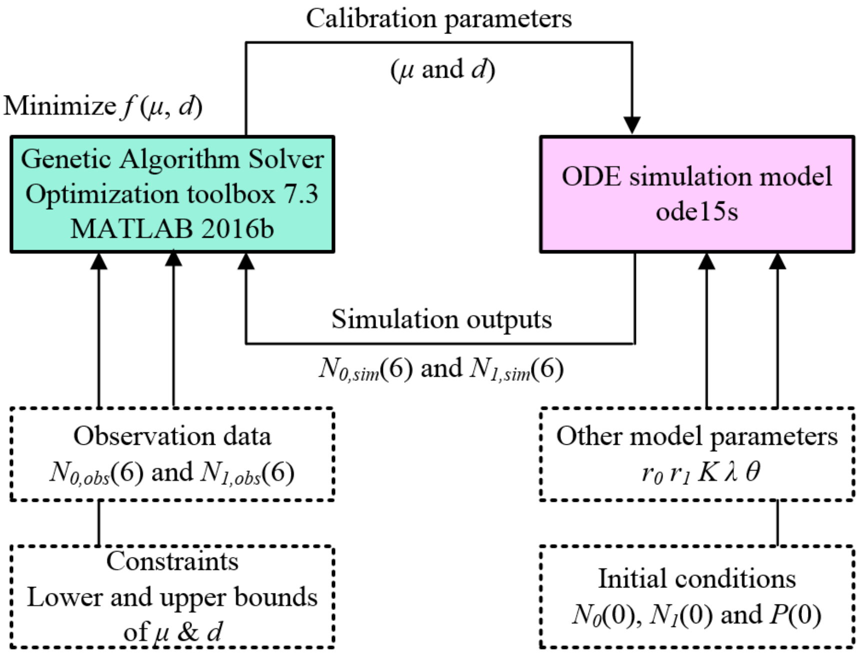

Fig. S1. Schematic diagram of the implicit calibration process

Table S2. Benchmark points of scale factor

Sequence number	1	2	3	4	5	6	7	8	9
Benchmark point of scale factor	0.05	0.1	0.2	0.5	1	2	5	10	20

Table S3. Variation range of K_{μ}

A	*B*	*C*	*D*	*E*	*F*	*G*	*H*
0.05-0.1	0.1-0.2	0.2-0.5	0.5-1	1-2	2-5	5-10	10-20

Table S4. Variation range of K_d

a	*b*	*c*	*d*	*e*	*f*	*g*	*h*
0.05-0.1	0.1-0.2	0.2-0.5	0.5-1	1-2	2-5	5-10	10-20

Table S5. Feasible subranges of the model calibration with two decision variables

$\Omega\mu$ \ Ωd	*a*	*b*	*c*	*d*	*e*	*f*	*g*	*h*
A	*A-a*	*A-b*	*A-c*	*A-d*	*A-e*	*A-f*	*A-g*	*A-h*
B	*B-a*	*B-b*	*B-c*	*B-d*	*B-e*	*B-f*	*B-g*	*B-h*
C	*C-a*	*C-b*	*C-c*	*C-d*	*C-e*	*C-f*	*C-g*	*C-h*
D	*D-a*	*D-b*	*D-c*	*D-d*	*D-e*	*D-f*	*D-g*	*D-h*
E	*E-a*	*E-b*	*E-c*	*E-d*	*E-e*	*E-f*	*E-g*	*E-h*
F	*F-a*	*F-b*	*F-c*	*F-d*	*F-e*	*F-f*	*F-g*	*F-h*
G	*G-a*	*G-b*	*G-c*	*G-d*	*G-e*	*G-f*	*G-g*	*G-h*
H	*H-a*	*H-b*	*H-c*	*H-d*	*H-e*	*H-f*	*H-g*	*H-h*

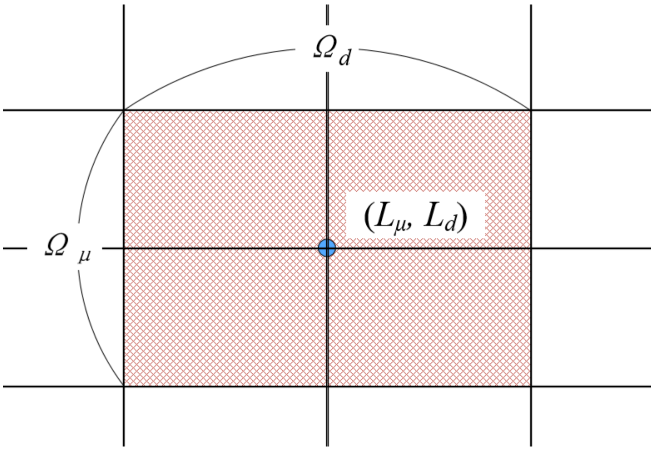

Fig. S2. Schematic diagram from point (L_1,L_2) to area Ω_μ-Ω_d.

The objective functions for searching benchmark points (L_μ, L_d) with different observation data can be written as:

$$\min \quad LS\left(R_\mu \cdot \mu_{ref}, R_d \cdot d_{ref}\right) = \alpha \cdot \left[N_{0,obs}(6) - N_{0,sim}(6)\right]^2 + \beta \cdot \left[N_{1,obs}(6) - N_{1,sim}(6)\right]^2$$

$$\begin{cases} R_\mu \in \Phi_\mu \\ R_d \in \Phi_d \\ \Phi_\mu = \Phi_d = \begin{Bmatrix} 0.05 & 0.1 & 0.2 & 0.5 & 1 \\ 2 & 5 & 10 & 20 & \end{Bmatrix} \end{cases} \quad \cdots\cdots \text{(S1)}$$

Parameters and descriptions are illustrated in Table S6.

Table S6. Parameters used in determining benchmark points (L_μ, L_d)

Parameter	Description
K_μ*	Optimal scale factor of transformation frequency
K_d*	Optimal scale factor of death rate
Ω_μ	Variation range of K_μ
L_μ	Benchmark point of K_μ, with the minimum value of LS function
A~H	Symbols of various Ω_μ
R_μ	Benchmark point of scale factor for transformation frequency
Φ	Set of benchmark points for scale factor
Ω_d	Variation range of K_d
L_d	Benchmark point of K_d, with the minimum value of LS function
a~h	Symbols of various Ω_d
R_d	Benchmark point of scale factor for death rate

The optimal benchmark point (L_μ, L_d) was calculated based on the observation data under different pharmaceutical-dosage conditions, and the feasible subranges (Ω_μ-Ω_d) of K_μ and K_d, as well as the upper and lower bounds of each decision variable were further determined (Table S7). Therefore, the optimal K_μ and K_d (i.e., K_μ*, K_d*) could be calculated based on the off-the-shelf Optimization Toolbox 7.3 in MATLAB 2016b.

Table S7. The optimal L_μ and L_d values, search ranges, upper and lower bounds under different conditions

Condition	L_μ	L_d	Ω_μ-Ω_d	LB_μ	UB_μ	LB_d	UB_d
Control	3	5	BC-de	0.1	0.5	0.5	2
Ibuprofen	5	5	DE-de	0.5	2	0.5	2
Naproxen	5	5	DE-de	0.5	2	0.5	2
Gemfibrozil	6	5	EF-de	1	5	0.5	2
Iopromide	3	5	BC-de	0.1	0.5	0.5	2
Diclofenac	5	5	DE-de	0.5	2	0.5	2
Propranolol	6	5	EF-de	1	5	0.5	2

Supplementary Figures

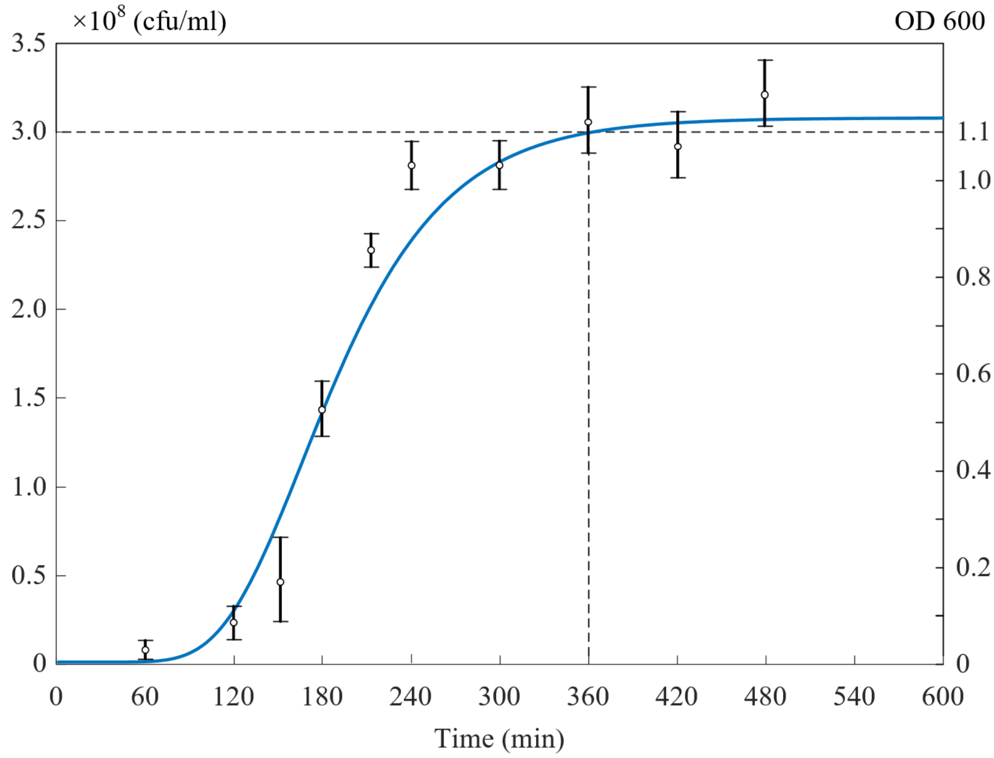

Fig. S3. Growth curve of *A. baylyi* ADP1 growing in 5 mL LB broth in a laid-down 50 mL Falcon tube at 30 ºC with 150 rpm shaking. The curve was simulated using the modified Gompertz model [9].

(a)

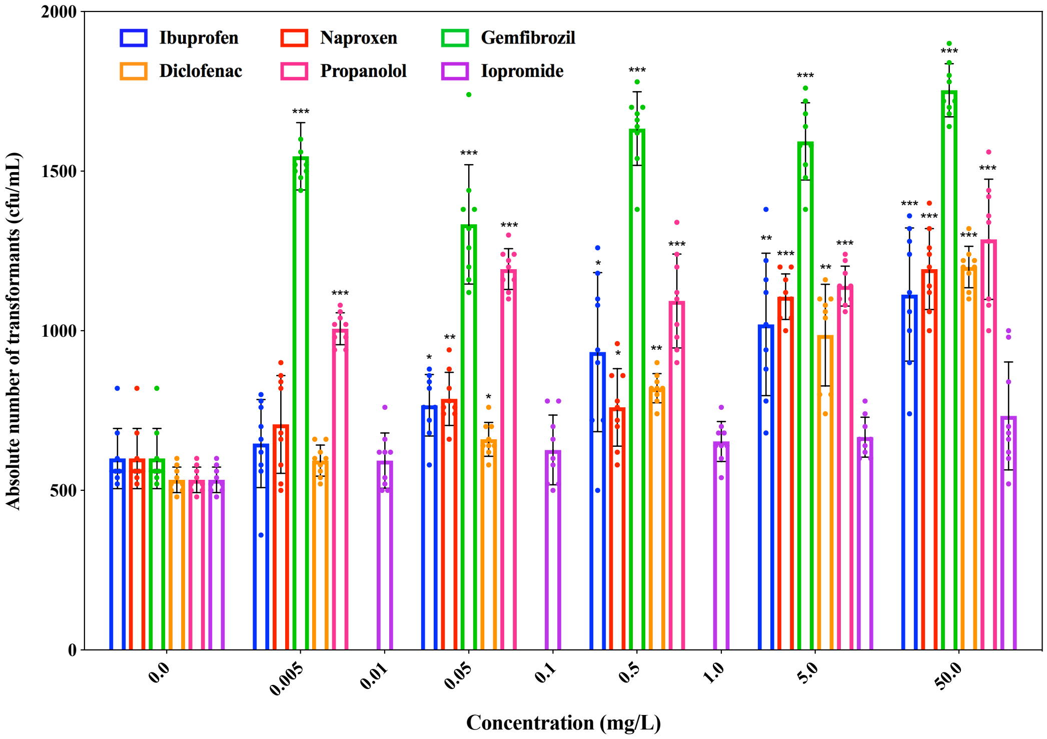
Ibuprofen
Naproxen
Gemfibrozil
Diclofenac
Propanolol
Iopromide
Absolute number of transformants (cfu/mL)
2000
1500
1000
500
0
0.0
0.005
0.01
0.05
0.1
0.5
1.0
5.0
50.0
Concentration (mg/L)

(b)

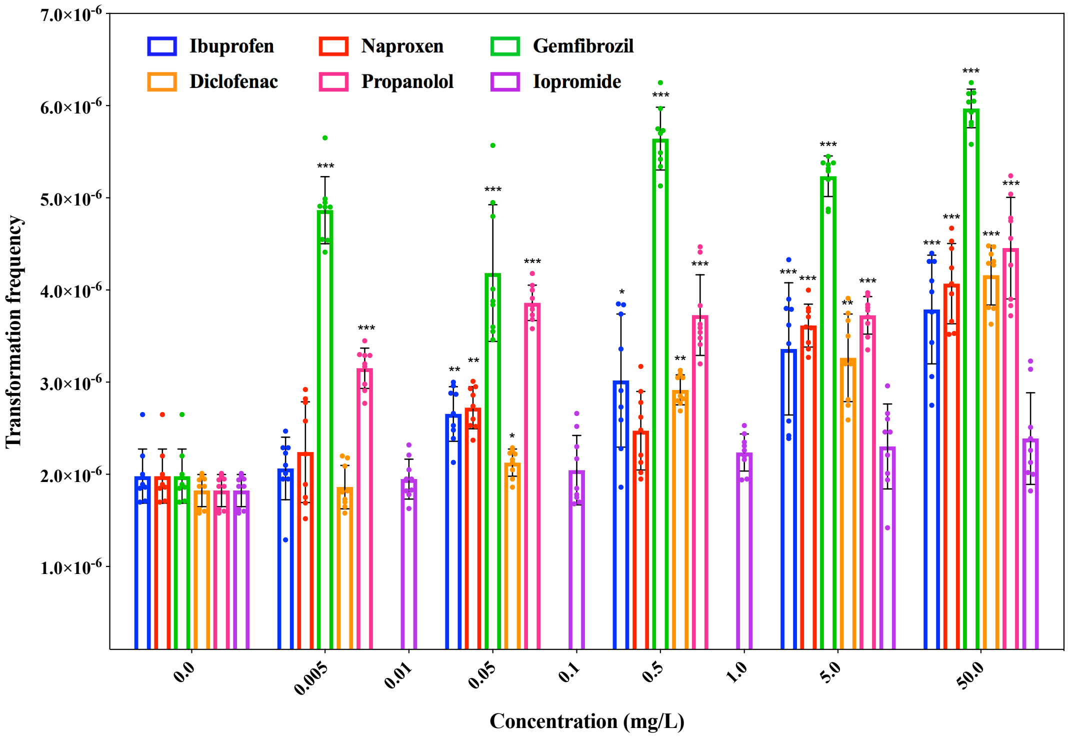
Ibuprofen
Naproxen
Gemfibrozil
Diclofenac
Propanolol
Iopromide
Transformation frequency
7.0×10⁻⁶
6.0×10⁻⁶
5.0×10⁻⁶
4.0×10⁻⁶
3.0×10⁻⁶
2.0×10⁻⁶
1.0×10⁻⁶
0.0
0.005
0.01
0.05
0.1
0.5
1.0
5.0
50.0
Concentration (mg/L)

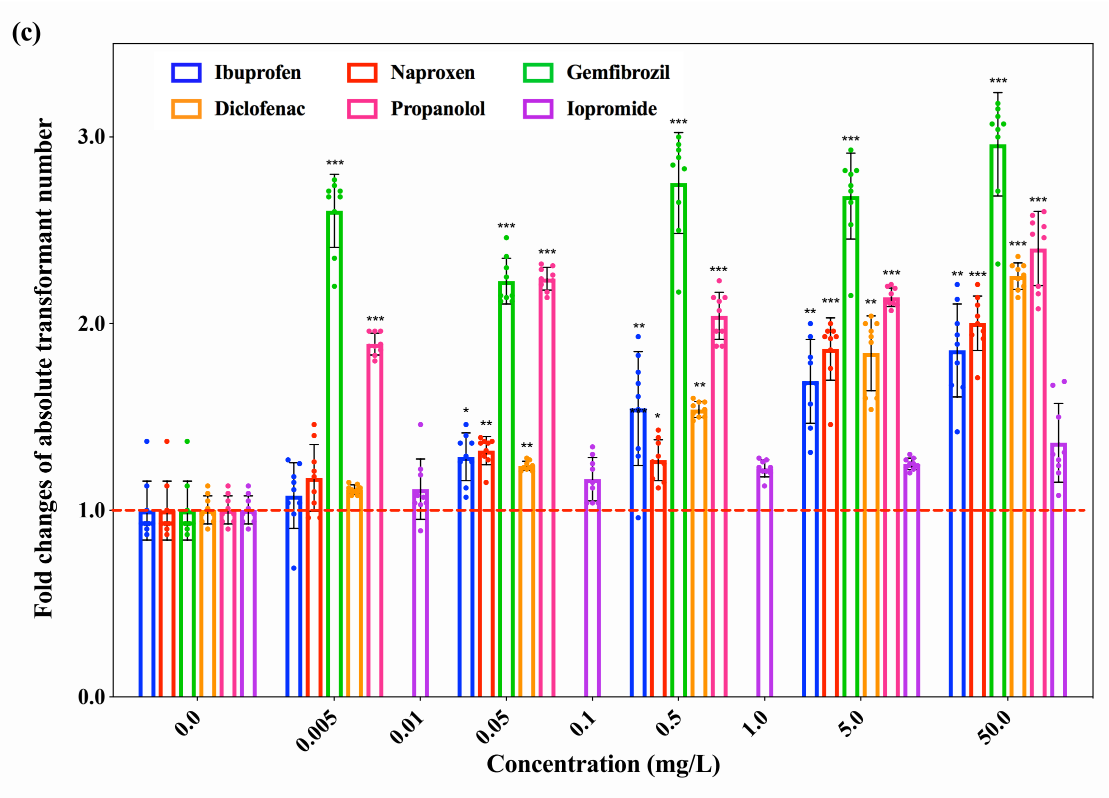

Fig. S4. Effects of non-antibiotic pharmaceuticals on transformation. (a) Absolute number of transformants. (b) Transformation frequency. (c) Fold changes of absolute transformant number, relative to pharmaceutical-free solvents. Significant differences between non-antibiotic-dosed samples and the control were analysed by independent-sample *t* test and corrected by Bonferroni correction method, * P^{*}<0.05, ** P^{*}<0.01, and *** P^{*}<0.001 (n=9).

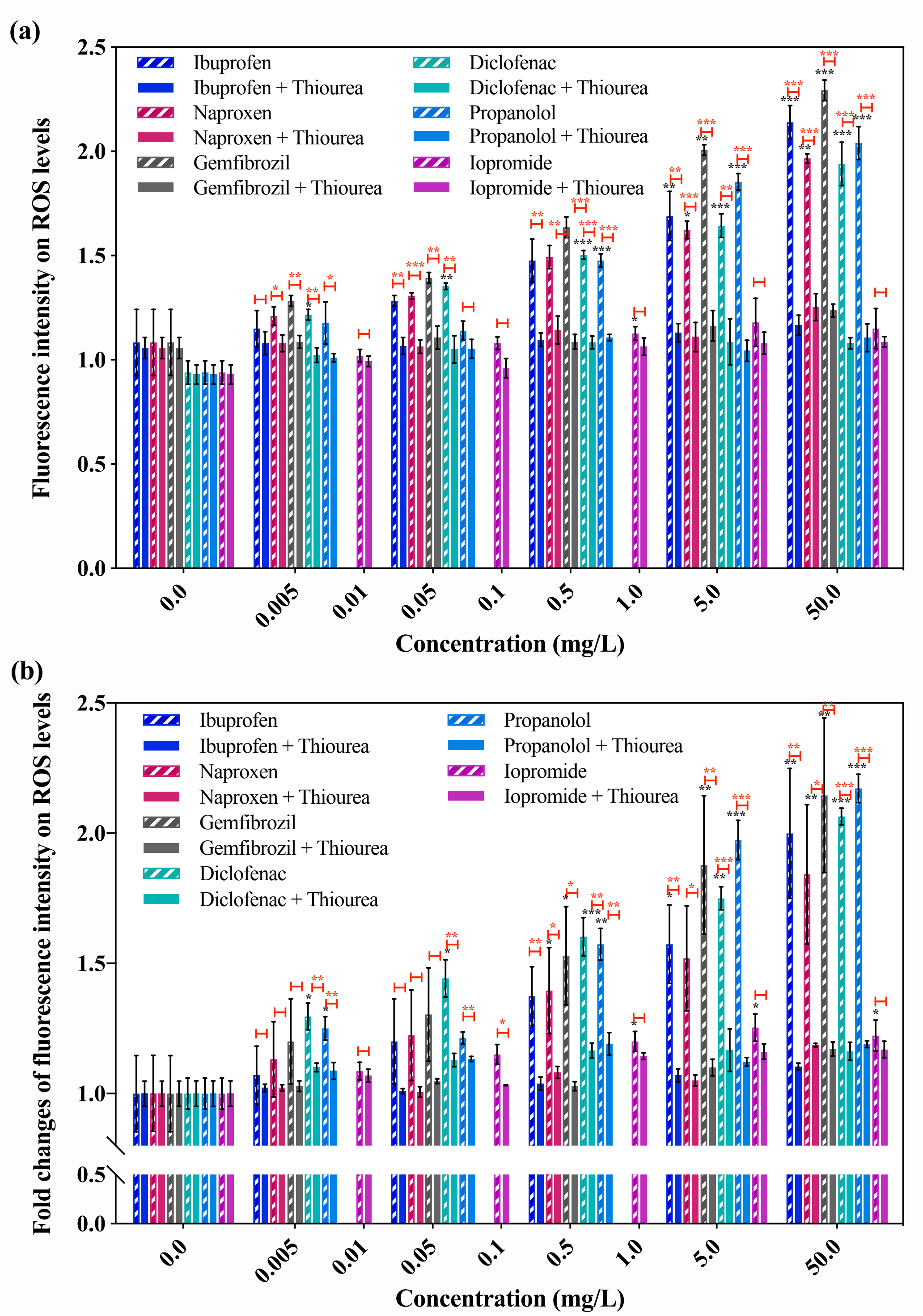

Fig. S5. Effects of non-antibiotic pharmaceuticals and thiourea on ROS of the bacteria *A. baylyi* ADP1. (a) Fluorescence intensity on ROS levels. (b) Fold changes of ROS generation. Significant differences between non-antibiotic-dosed samples and the control were analysed by independent-sample *t* test and corrected by Bonferroni correction method, * $P^{*}<0.05$, ** $P^{*}<0.01$, and *** $P^{*}<0.001$.

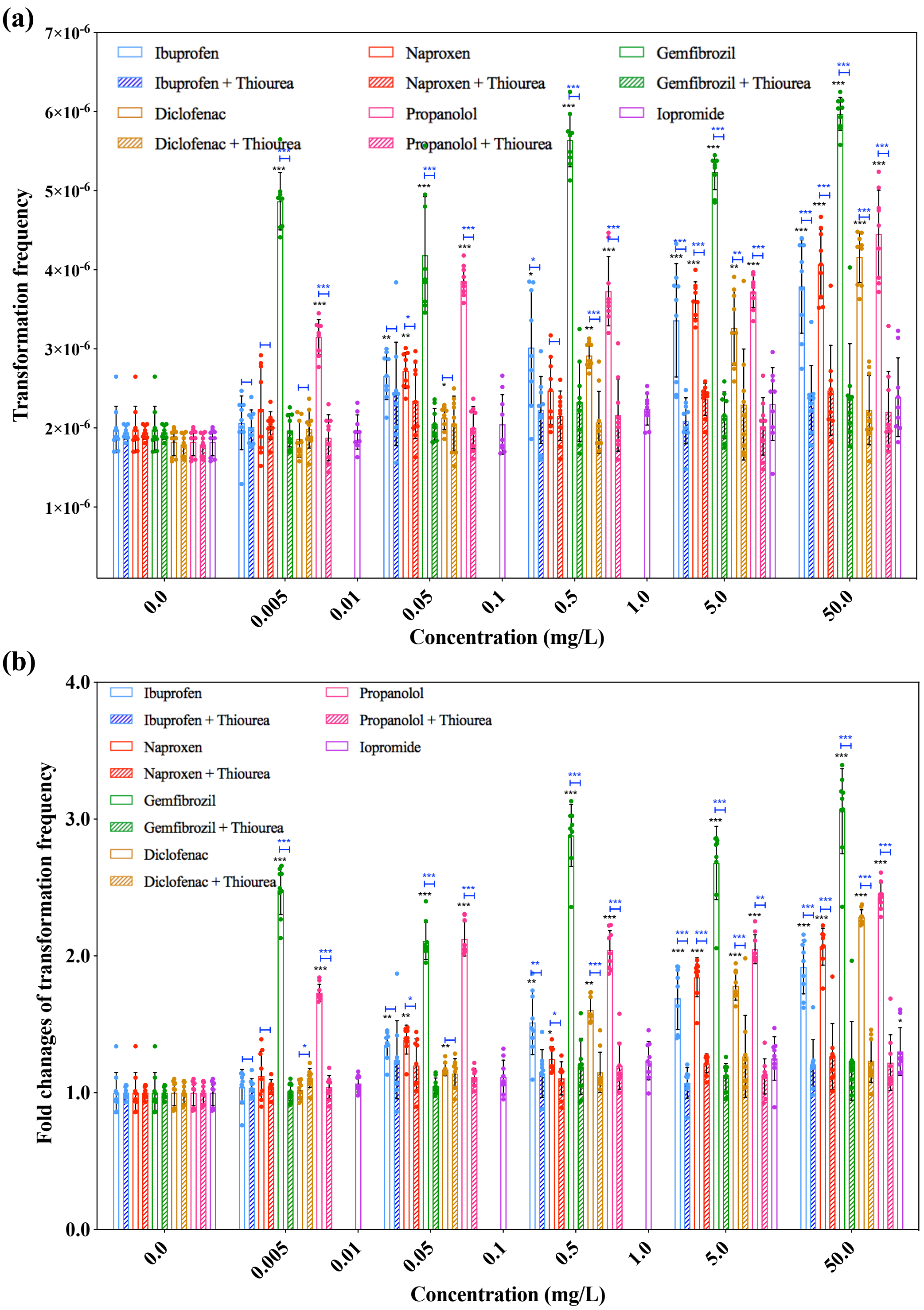

Fig. S6. Effects of non-antibiotic pharmaceuticals and thiourea on transformation of free pWH1266 plasmid to *A. baylyi* ADP1. (a) Transformation frequency with the addition of ROS scavenger thiourea. (b) Fold changes of transformation frequency with the addition of ROS scavenger thiourea. Significant differences between non-antibiotic-dosed samples and the control were analysed by independent-sample *t* test and corrected by Bonferroni correction method, * $P^{*}<0.05$, ** $P^{*}<0.01$, and *** $P^{*}<0.001$ (n=9).

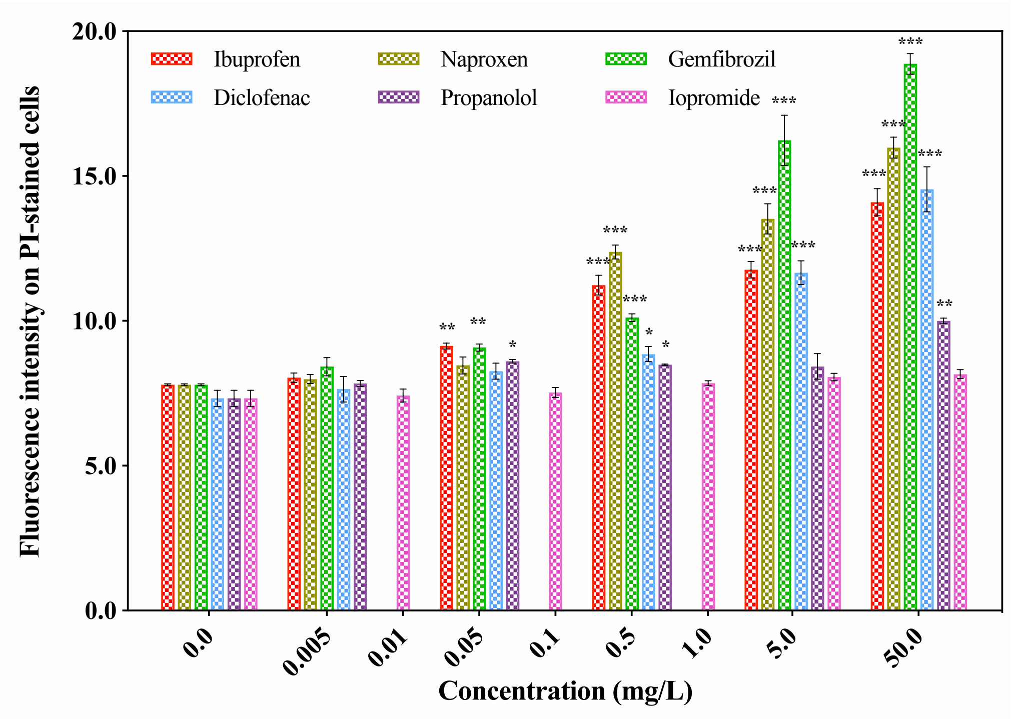

Fig. S7. Effects of non-antibiotic pharmaceuticals on cell membrane permeability of the bacteria *A. baylyi* ADP1. Fluorescence intensity on PI-stained cells. Significant differences between non-antibiotic-dosed samples and the control were analysed by independent-sample *t* test and corrected by Bonferroni correction method, * $P^{*}<0.05$, ** $P^{*}<0.01$, and *** $P^{*}<0.001$.

Supplementary Tables

Table S8. Concentrations of non-antibiotic pharmaceuticals in various environmental settings

Non-antibiotic pharmaceutical	Municipal wastewater treatment plant[1]		Hospital wastewater (μg/L)[1]	Surface water (ng/L, include river, stream, lake)	References
	Influent concentration (μg/L)	Effluent concentration (μg/L)			
Ibuprofen	0.1-1000	0.001-100	1.5-151	7.7, 7.8-80, 10-1000	[10-17]
Naproxen	0.1-100	0.001-50	0.01-21.8	10-380, 10-1000	[10-12,14,16,18]
Gemfibrozil	0.5-100	0.01-10	1.1-7.3	510, 10-1000	[10,14,18-20]
Diclofenac	0.1-50	0.01-10	0.028-73	10-140, 1200, 10-1000	[10-12,14,16-21]
Propranolol	0.01-50	0.01-5	0.2-6.5	590, 10-1000	[10,14,17,20,22,23]
Iopromide	0.01-10	0.01-10	14.3-326.9	100-910	[10,11,24,25]

Note:

1. Ibuprofen, naproxen, and diclofenac are over the counter (OTC) drugs, while gemfibrozil and propranolol are available on prescription. These five drugs are mostly consumed in households. Iopromide, as a contrast media, is mostly consumed in hospitals.

Table S9. Concentrations of non-antibiotic pharmaceuticals in clinical setting

Non-antibiotic pharmaceutical	Dose (mg/day)	Plasma concentration (μg/L)	Excretion mode	References
Ibuprofen	800-3200	21300-60000	Metabolic, 0%-3% excreted in urine unchanged	[26-29]
Naproxen	500-1000	22000-80000	Metabolic, 20% excreted in urine unchanged	[30,31]
Gemfibrozil	1200	30300-61800	Metabolic, 0.02%-0.2% excreted in urine unchanged, feces 6%	[32,33]
Diclofenac	100-150	20-2206	Metabolic, 4.4%-8% excreted in urine unchanged	[34,35]
Propranolol	80-640	5.3-300	Metabolic, 0%-3% excreted in urine unchanged	[36-38]
Iopromide	150-300 mg/kg	Not applicable	Non-metabolic, 36.6%-56.2% excreted in urine unchanged	[39]

Table S10. Minimum inhibitory concentrations (MICs) of strain *A. baylyi* ADP1 towards non-antibiotic pharmaceuticals

Strain	MICs (mg/L)					
	Ibuprofen	Naproxen	Gemfibrozil	Iopromide	Diclofenac	Propranolol
A. baylyi ADP1	1000	1000	500	>50	500	500

Table S11. Transformation results under the exposure of non-antibiotic pharmaceuticals for 6 h *

	Concentration (mg/L)	Ibuprofen	Naproxen	Gemfibrozil	Diclofenac	Propranolol	Iopromide #
Absolute transformant (cfu/mL)	0	600.0±88.9	600.0±88.9	600.0±88.9	533.3±37.7	533.3±37.7	533.3±37.7
	0.005	646.7±130.0	706.7±144.5	1546.7±99.3	593.3±46.2	1006.7±47.1	593.3±81.6
	0.05	766.7±90.9	786.7±78.3	1333.3±176.4	660.0±49.9	1193.3±60.4	626.7±102.8
	0.5	933.3±243.9	760.0±114.3	1633.3±108.7	820.0±43.2	1093.3±138.9	653.3±58.9
	5.0	1020.0±210.4	1106.7±67.3	1593.3±114.3	986.7±150.3	1140.0±58.9	666.7±58.9
	50.0	1113.3±196.6	1193.3±119.6	1753.3±78.3	1200.0±61.1	1286.7±177.6	733.3±159.4
Total viable bacteria (cfu/mL)	0	$3.0\times10^{8}\pm5.1\times10^{6}$	$3.0\times10^{8}\pm5.1\times10^{6}$	$3.0\times10^{8}\pm5.1\times10^{6}$	$2.9\times10^{8}\pm2.2\times10^{7}$	$2.9\times10^{8}\pm2.2\times10^{7}$	$2.9\times10^{8}\pm2.2\times10^{7}$
	0.005	$3.1\times10^{8}\pm8.2\times10^{6}$	$3.2\times10^{8}\pm1.6\times10^{7}$	$3.2\times10^{8}\pm2.0\times10^{7}$	$3.2\times10^{8}\pm2.2\times10^{7}$	$3.2\times10^{8}\pm2.1\times10^{7}$	$3.0\times10^{8}\pm1.2\times10^{7}$
	0.05	$2.9\times10^{8}\pm9.0\times10^{6}$	$2.9\times10^{8}\pm6.4\times10^{6}$	$3.2\times10^{8}\pm1.7\times10^{7}$	$3.1\times10^{8}\pm6.4\times10^{6}$	$3.1\times10^{8}\pm1.0\times10^{7}$	$3.1\times10^{8}\pm1.1\times10^{7}$
	0.5	$3.1\times10^{8}\pm3.0\times10^{6}$	$3.2\times10^{8}\pm2.1\times10^{7}$	$2.9\times10^{8}\pm1.0\times10^{7}$	$2.8\times10^{8}\pm1.2\times10^{7}$	$2.9\times10^{8}\pm1.1\times10^{7}$	$2.9\times10^{8}\pm1.2\times10^{7}$
	5.0	$3.0\times10^{8}\pm8.2\times10^{6}$	$3.1\times10^{8}\pm5.1\times10^{6}$	$3.0\times10^{8}\pm1.1\times10^{7}$	$3.0\times10^{8}\pm1.5\times10^{7}$	$3.1\times10^{8}\pm2.6\times10^{7}$	$3.0\times10^{8}\pm1.6\times10^{7}$
	50.0	$2.9\times10^{8}\pm1.3\times10^{7}$	$2.9\times10^{8}\pm2.6\times10^{6}$	$2.9\times10^{8}\pm1.1\times10^{7}$	$2.9\times10^{8}\pm1.8\times10^{7}$	$2.9\times10^{8}\pm9.0\times10^{6}$	$3.1\times10^{8}\pm9.2\times10^{6}$
Transformation frequency	0	$2.0\times10^{-6}\pm2.8\times10^{-7}$	$2.0\times10^{-6}\pm2.8\times10^{-7}$	$2.0\times10^{-6}\pm2.8\times10^{-7}$	$1.8\times10^{-6}\pm1.6\times10^{-7}$	$1.8\times10^{-6}\pm1.6\times10^{-7}$	$1.8\times10^{-6}\pm1.6\times10^{-7}$
	0.005	$2.1\times10^{-6}\pm3.2\times10^{-7}$	$2.2\times10^{-6}\pm5.2\times10^{-7}$	$4.9\times10^{-6}\pm3.4\times10^{-7}$	$1.9\times10^{-6}\pm2.2\times10^{-7}$	$3.2\times10^{-6}\pm2.1\times10^{-7}$	$1.9\times10^{-6}\pm2.0\times10^{-7}$
	0.05	$2.7\times10^{-6}\pm2.8\times10^{-7}$	$2.7\times10^{-6}\pm2.1\times10^{-7}$	$4.2\times10^{-6}\pm7.0\times10^{-7}$	$2.1\times10^{-6}\pm1.4\times10^{-7}$	$3.9\times10^{-6}\pm1.8\times10^{-7}$	$2.0\times10^{-6}\pm3.5\times10^{-7}$
	0.5	$3.0\times10^{-6}\pm6.8\times10^{-7}$	$2.5\times10^{-6}\pm4.0\times10^{-7}$	$5.6\times10^{-6}\pm3.2\times10^{-7}$	$2.9\times10^{-6}\pm1.5\times10^{-7}$	$3.7\times10^{-6}\pm4.1\times10^{-7}$	$2.2\times10^{-6}\pm1.9\times10^{-7}$
	5.0	$3.4\times10^{-6}\pm6.7\times10^{-7}$	$3.5\times10^{-6}\pm2.2\times10^{-7}$	$5.2\times10^{-6}\pm2.1\times10^{-7}$	$3.3\times10^{-6}\pm4.5\times10^{-7}$	$3.7\times10^{-6}\pm1.9\times10^{-7}$	$2.3\times10^{-6}\pm4.4\times10^{-7}$
	50.0	$3.8\times10^{-6}\pm5.6\times10^{-7}$	$4.1\times10^{-6}\pm4.1\times10^{-7}$	$6.0\times10^{-6}\pm2.0\times10^{-7}$	$4.2\times10^{-6}\pm3.0\times10^{-7}$	$4.5\times10^{-6}\pm5.2\times10^{-7}$	$2.4\times10^{-6}\pm4.7\times10^{-7}$
Fold change of absolute transformant number	0.005	1.08±0.16	1.18±0.17	2.60±0.19	1.11±0.02	1.89±0.06	1.11±0.15
	0.05	1.29±0.12	1.32±0.07	2.23±0.12	1.24±0.02	2.24±0.06	1.17±0.11
	0.5	1.54±0.29	1.27±0.10	2.76±0.25	1.54±0.04	2.04±0.12	1.22±0.04
	5.0	1.69±0.21	1.87±0.16	2.68±0.22	1.84±0.19	2.14±0.05	1.25±0.03
	50.0	1.86±0.24	2.00±0.14	2.96±0.26	2.25±0.07	2.40±0.19	1.36±0.20
Fold change of transformation frequency	0.005	1.05±0.12	1.12±0.17	2.48±0.17	1.02±0.06	1.73±0.06	1.07±0.06
	0.05	1.35±0.10	1.39±0.10	2.11±0.13	1.17±0.04	2.13±0.12	1.11±0.12
	0.5	1.51±0.22	1.25±0.09	2.88±0.21	1.61±0.08	2.04±0.13	1.24±0.13
	5.0	1.69±0.22	1.84±0.13	2.68±0.25	1.78±0.10	2.05±0.10	1.25±0.15
	50.0	1.92±0.19	2.05±0.13	3.04±0.29	2.28±0.05	2.43±0.09	1.30±0.16

* n=9, data are shown as mean ± SD, fold changes were in comparison with the corresponding control values.
# The concentrations for iopromide are 0.01, 0.1, 1, 5, 50 mg/L, respectively

Table S12. Minimum inhibitory concentrations (MICs) of donor, recipient, and different transformants towards antibiotics*

Antibiotics	MICs (mg/L)									
	E. coli harbouring pWH1266 plasmid	Recipient	TM 1	TM 2	TM 3	TM 4	TM 5	TM 6	TM 7	TM 8
Tetracycline	32	4	32	32	32	32	32	32	32	32
Ampicillin	256	64	256	256	256	256	256	256	256	256

* TM 1-8: transformants in transformation system treated with Milli-Q water, ethanol, ibuprofen, naproxen, gemfibrozil, diclofenac, propranolol, iopromide, respectively

Table S13. Genes relevant to ROS production in *A. baylyi* ADP1 after exposure of non-antibiotic pharmaceuticals

**Gene**	**COG Annotation**	**Fold Change of FPKM** *					
		**Ibuprofen**	**Naproxen**	**Gemfibrozil**	**Diclofenac**	**Propranolol**	**Iopromide**
ahpC	peroxiredoxin	1.13	1.40	1.37	1.05	1.24	1.00
ahpF	alkyl hydroperoxide reductase subunit F	1.03	1.16	1.15	0.98	1.43	0.96
alkB	alpha-ketoglutarate-dependent dioxygenase AlkB	1.14	1.76	1.28	1.57	1.48	0.98
alkK	long-chain-fatty-acid--CoA ligase	2.93	1.04	1.32	2.74	2.77	0.98
alkM	alkane 1-monooxygenase	2.58	2.26	2.23	0.82	1.17	0.89
alkR	AraC family transcriptional regulator	2.45	2.52	1.56	1.16	1.26	1.38
bfr	regulatory or redox protein complexing with Bfr in iron storage and mobility (BFD)	1.27	5.47	0.61	1.57	2.17	1.35
estR	hydrogen peroxide-inducible genes activator	1.15	1.12	1.17	1.26	1.41	0.89

Gene	COG Annotation	Fold Change of FPKM *					
		Ibuprofen	Naproxen	Gemfibrozil	Diclofenac	Propranolol	Iopromide
fdhF	FdhF/YdeP family oxidoreductase	1.03	0.89	1.12	1.33	1.21	1.30
hipA	type II toxin-antitoxin system HipA family toxin	1.32	1.85	1.33	1.50	1.11	0.87
mdaB	NAD(P)H-dependent oxidoreductase	1.02	1.07	1.14	1.58	1.31	1.61
msrA	peptide-methionine (S)-S-oxide reductase MsrA	1.39	1.41	1.50	1.92	2.10	1.72
sodA	superoxide dismutase [Mn]	1.69	1.38	1.38	0.61	0.56	0.51
sodB	superoxide dismutase	1.10	1.21	1.18	1.05	1.47	0.86
sodM	superoxide dismutase	1.41	0.82	0.86	1.56	1.08	1.04
soxA	FAD-dependent oxidoreductase	1.52	0.93	1.30	1.12	1.22	0.95
soxB	FAD-dependent oxidoreductase	0.90	1.01	1.11	1.58	1.17	1.00
soxD	sarcosine oxidase subunit delta	3.09	1.55	2.06	0.97	3.48	0.48

Gene	COG Annotation	Fold Change of FPKM *					
		Ibuprofen	Naproxen	Gemfibrozil	Diclofenac	Propranolol	Iopromide
soxR	redox-sensitive transcriptional activator SoxR	2.38	1.58	1.20	1.24	0.93	0.68
trxB	thioredoxin-disulfide reductase	1.18	1.04	1.24	1.19	1.45	0.94
ychF	redox-regulated ATPase YchF	1.27	0.80	0.68	1.52	1.96	0.69
ACIAD0019	NAD(P)H-dependent oxidoreductase	1.38	1.99	1.67	1.10	1.08	0.90
ACIAD0282	oxidative damage protection protein	0.87	1.16	1.02	1.12	1.73	1.03
ACIAD1733	NAD(P)/FAD-dependent oxidoreductase	1.69	1.07	1.93	1.64	1.38	1.39
ACIAD2104	SDR family oxidoreductase	1.39	1.80	1.73	1.54	1.94	1.76
ACIAD2339	NAD(P)/FAD-dependent oxidoreductase	7.41	6.79	5.84	0.86	0.67	0.75
ACIAD2570	SDR family NAD(P)-dependent oxidoreductase	1.83	2.39	3.04	2.40	1.57	2.50

Gene	COG Annotation	Fold Change of FPKM *					
		Ibuprofen	Naproxen	Gemfibrozil	Diclofenac	Propranolol	Iopromide
ACIAD2794	NAD(P)/FAD-dependent oxidoreductase	1.42	1.18	1.84	1.24	0.50	1.27
ACIAD4510	oxidoreductase	1.63	1.39	1.99	0.71	1.46	0.30
ACIAD4555	SDR family oxidoreductase	1.29	2.42	4.87	2.09	1.54	0.93
ACIAD4740	oxygen-dependent coproporphyrinogen oxidase	1.04	1.09	1.24	1.34	2.19	1.25

*: Comparing with the control group without pharmaceutical dosage

Table S14. Proteins relevant to ROS production in *A. baylyi* ADP1 after exposure of non-antibiotic pharmaceuticals

Protein	Description	Fold Change of Protein Abundance *					
		**Ibuprofen**	**Naproxen**	**Gemfibrozil**	**Diclofenac**	**Propranolol**	**Iopromide**
AhpC	Alkyl hydroperoxide reductase, C22 subunit, thioredoxin-like (Detoxification of hydroperoxides)	0.87	0.88	1.12	1.39	1.27	0.85
AhpF	Alkyl hydroperoxide reductase subunit, FAD/NAD(P)-binding, detoxification of hydroperoxides	1.20	1.13	1.29	1.10	1.13	1.10
Bfr	Bacterioferritin	1.72	0.80	0.84	1.14	1.54	0.74
SodA	Superoxide dismutase [Mn]	2.36	2.69	2.22	1.21	2.50	0.49
SodB	Superoxide dismutase	2.15	1.63	2.23	1.80	1.56	1.30
TrxA	Thioredoxin	6.48	5.54	6.69	0.67	1.97	0.44
TrxB	Thioredoxin reductase	0.98	0.92	0.98	1.55	1.21	0.96
YchF	Ribosome-binding ATPase YchF	1.10	0.95	1.07	1.59	1.88	0.96

*: Comparing with the control group without pharmaceutical dosage

Table S15. Genes relevant to stress response in *A. baylyi* ADP1 after exposure of non-antibiotic pharmaceuticals

**Gene**	**COG Annotation**	**Fold Change of FPKM [*]**					
		**Ibuprofen**	**Naproxen**	**Gemfibrozil**	**Diclofenac**	**Propranolol**	**Iopromide**
glsB	GlsB/YeaQ/YmgE family stress response membrane protein	1.39	1.58	1.47	0.53	0.81	0.45
nirD	NirD/YgiW/YdeI family stress tolerance protein	0.77	1.15	1.14	1.13	1.08	1.38
umuD	translesion error-prone DNA polymerase V autoproteolytic subunit	0.96	1.12	1.21	0.95	1.47	0.87
yaaA	peroxide stress protein YaaA	2.04	1.25	1.77	1.21	1.17	1.36
ygiW	NirD/YgiW/YdeI family stress tolerance protein	1.53	1.02	1.20	0.77	1.59	0.67
ACIAD1238	universal stress protein	1.05	0.99	1.11	1.76	0.65	0.50
ACIAD1493	universal stress protein	1.82	0.88	1.12	0.92	0.89	1.09
ACIAD2005	universal stress protein	2.10	1.84	1.14	1.54	1.10	1.00
ACIAD2863	universal stress protein	1.21	1.69	1.16	0.77	1.41	0.81
ACIAD2865	universal stress protein	1.99	0.82	1.65	1.39	1.35	0.65

*: Comparing with the control group without pharmaceutical dosage

Table S16. Proteins relevant to stress response in *A. baylyi* ADP1 after exposure of non-antibiotic pharmaceuticals

Protein	Description	Fold Change of Protein Abundance *					
		Ibuprofen	Naproxen	Gemfibrozil	Diclofenac	Propranolol	Iopromide
ACIAD2005	universal stress protein	5.38	5.43	2.09	9.21	10.48	3.06

*: Comparing with the control group without pharmaceutical dosage

Table S17. Genes relevant to cell membrane in *A. baylyi* ADP1 after exposure of non-antibiotic pharmaceuticals

Gene	COG Annotation	Fold Change of FPKM *					
		**Ibuprofen**	**Naproxen**	**Gemfibrozil**	**Diclofenac**	**Propranolol**	**Iopromide**
acuC	thin pilus assembly outer membrane usher AcuC	1.73	0.92	1.01	0.67	0.56	0.75
atpI	ATP synthase subunit I	1.77	0.60	0.62	1.85	1.06	0.50
bamA	outer membrane protein assembly factor BamA	1.54	1.12	1.25	1.03	1.14	0.96
bamB	outer membrane protein assembly factor BamB	1.52	0.96	0.96	1.04	1.18	1.03
bamD	outer membrane protein assembly factor BamD	1.20	1.34	0.85	1.12	1.43	0.89
bamE	outer membrane protein assembly factor BamE	2.58	1.25	1.49	2.06	2.15	1.02
hcaE	OprD family porin	1.17	1.35	1.07	1.53	2.09	1.62
lolB	outer membrane lipoprotein LolB	1.24	1.22	1.21	1.03	1.02	0.99
ompH	OmpH family outer membrane protein	1.07	1.04	1.37	1.35	1.01	1.06

Gene	COG Annotation	Fold Change of FPKM *					
		Ibuprofen	Naproxen	Gemfibrozil	Diclofenac	Propranolol	Iopromide
ompR	two-component system response regulator OmpR	1.02	1.00	1.03	1.14	0.97	0.86
oprD	OprD family porin	1.53	0.73	0.75	1.69	2.95	0.73
smpA	outer membrane protein assembly factor BamE	0.99	1.45	1.02	1.51	1.18	0.95
tolC	TolC family outer membrane protein	1.56	1.14	1.07	1.55	1.33	1.01
vacJ	VacJ family lipoprotein	1.13	1.12	1.18	0.93	1.09	0.82
ACIAD0111	membrane protein	2.89	4.42	3.97	2.66	2.81	2.75
ACIAD0610	porin	3.08	3.22	3.97	1.40	1.51	1.46
ACIAD0799	membrane protein	0.88	0.76	0.68	2.64	1.96	2.27
ACIAD0898	membrane protein	1.05	1.20	1.26	0.49	0.80	0.45
ACIAD1160	efflux transporter outer membrane subunit	0.91	1.05	1.71	1.66	1.28	0.94
ACIAD1924	membrane protein	1.13	1.00	1.31	1.16	1.21	1.01

Gene	COG Annotation	Fold Change of FPKM [*]					
		**Ibuprofen**	**Naproxen**	**Gemfibrozil**	**Diclofenac**	**Propranolol**	**Iopromide**
ACIAD2246	porin	1.50	1.35	0.79	1.35	0.56	0.93
ACIAD2403	outer membrane protein assembly factor	1.16	1.10	1.24	0.77	0.70	0.89
ACIAD2984	carbohydrate porin, cell outer membrane; pore complex	1.41	1.64	1.60	1.17	1.77	1.16
ACIAD3499	putative porin	1.11	1.06	1.13	2.96	1.99	0.96
ACIAD6460	TIGR04219 family outer membrane beta-barrel protein	1.42	0.77	1.50	1.18	1.38	0.85

[*]: Comparing with the control group without pharmaceutical dosage

Table S18. Proteins relevant to cell membrane in *A. baylyi* ADP1 after exposure of non-antibiotic pharmaceuticals

**Protein**	**Description**	**Fold Change of Protein Abundance** *					
		**Ibuprofen**	**Naproxen**	**Gemfibrozil**	**Diclofenac**	**Propranolol**	**Iopromide**
AdeC	Outer membrane protein (AdeC-like)	2.06	1.90	2.30	1.18	1.83	2.06
BamA	Outer membrane protein assembly factor BamA	1.10	1.11	1.19	1.00	1.17	0.97
BamD	Outer membrane protein assembly factor BamD	1.10	1.13	1.26	0.76	0.95	1.10
TolB	Tol-Pal system protein TolB	1.78	1.71	1.31	1.81	1.94	0.74

*: Comparing with the control group without pharmaceutical dosage

Table S19. Proteins relevant to DNA repair and recombination in *A. baylyi* ADP1 after exposure of non-antibiotic pharmaceuticals

Protein	Description	Fold Change of Protein Abundance *					
		Ibuprofen	Naproxen	Gemfibrozil	Diclofenac	Propranolol	Iopromide
GyrB	DNA gyrase subunit B	1.83	1.90	1.47	1.06	1.49	1.79
HimA	Integration host factor subunit alpha	1.15	1.04	1.12	1.12	0.86	1.08
Ssb	Single-stranded DNA-binding protein	1.15	1.08	0.86	1.24	1.85	0.95

*: Comparing with the control group without pharmaceutical dosage

Table S20. Genes relevant to DNA repair and recombination in *A. baylyi* ADP1 after exposure of non-antibiotic pharmaceuticals

Gene	COG Annotation	Fold Change of FPKM *					
		**Ibuprofen**	**Naproxen**	**Gemfibrozil**	**Diclofenac**	**Propranolol**	**Iopromide**
dinB	DNA damage-inducible protein DinB	1.93	2.92	2.54	1.99	1.41	1.21
gyrA	DNA gyrase subunit A	0.85	0.96	1.03	1.12	1.19	0.97
gyrB	DNA topoisomerase (ATP-hydrolyzing) subunit B	1.01	1.06	1.13	1.07	1.38	1.11
himA	integration host factor subunit alpha	1.08	1.17	0.96	0.74	1.15	0.90
himD	integration host factor subunit beta	1.20	1.74	1.55	0.85	0.90	0.68
parC	DNA topoisomerase IV subunit A	0.89	1.06	0.95	1.03	1.43	0.95
parE	DNA topoisomerase IV subunit B	0.87	0.83	1.00	1.41	1.85	1.39
recA	recombinase RecA	1.09	1.00	1.03	0.91	1.07	0.94
recB	exonuclease V subunit beta	1.24	0.95	1.13	1.22	1.16	0.96

Gene	COG Annotation	Fold Change of FPKM *					
		Ibuprofen	Naproxen	Gemfibrozil	Diclofenac	Propranolol	Iopromide
recC	exonuclease V subunit gamma	1.13	0.98	1.03	1.15	1.01	0.79
recD	exodeoxyribonuclease V subunit alpha, DNA metabolism	1.28	1.14	1.13	1.11	1.03	1.38
recF	DNA replication/repair protein RecF	1.03	0.95	1.10	1.12	0.89	1.03
recN	DNA repair protein RecN	0.87	1.29	0.87	1.09	1.27	1.01
recO	DNA repair protein RecO	1.26	0.99	1.27	1.17	1.20	1.26
recR	recombination protein RecR	1.11	1.08	1.23	1.16	1.40	1.16
ssb	single-stranded DNA-binding protein	1.21	0.67	0.76	0.88	1.13	0.68
uvrB	excinuclease ABC subunit UvrB	1.16	1.18	1.18	1.05	1.25	0.99

*: Comparing with the control group without pharmaceutical dosage

Table S21. Genes relevant to T6SS in *A. baylyi* ADP1 after exposure of non-antibiotic pharmaceuticals

Gene	COG Annotation	Fold Change of FPKM *					
		Ibuprofen	Naproxen	Gemfibrozil	Diclofenac	Propranolol	Iopromide
vgrG	type VI secretion system tip protein VgrG	1.37	1.41	1.40	2.04	0.52	0.54
ACIAD0167	type VI secretion system tip protein VgrG	1.47	1.49	1.21	0.78	1.48	0.91
ACIAD3427	type VI secretion system tip protein VgrG	0.86	0.85	1.05	1.08	1.07	1.23

*: Comparing with the control group without pharmaceutical dosage

Table S22. Genes relevant to efflux pump in *A. baylyi* ADP1 after exposure of non-antibiotic pharmaceuticals

**Gene**	**COG Annotation**	**Fold Change of FPKM** *					
		**Ibuprofen**	**Naproxen**	**Gemfibrozil**	**Diclofenac**	**Propranolol**	**Iopromide**
acrR	TetR/AcrR family transcriptional regulator	1.15	1.26	1.22	1.63	1.44	1.47
aceI	chlorhexidine efflux PACE transporter AceI	1.38	1.33	0.93	0.92	3.89	0.91
hcaR	MarR family transcriptional regulator	1.84	0.60	1.17	2.16	1.81	0.81
marR	MarR family transcriptional regulator	2.10	3.03	3.83	3.12	2.93	1.72
tetR	TetR/AcrR family transcriptional regulator	2.04	3.23	2.06	2.62	1.07	0.78
ACIAD0026	TetR family transcriptional regulator	1.08	1.22	1.12	1.30	1.60	1.09
ACIAD0217	TetR/AcrR family transcriptional regulator	1.53	1.82	1.62	1.30	1.50	0.92

Gene	COG Annotation	Fold Change of FPKM *					
		Ibuprofen	Naproxen	Gemfibrozil	Diclofenac	Propranolol	Iopromide
ACIAD0504	TetR/AcrR family transcriptional regulator	1.65	1.09	1.35	0.93	1.49	1.45
ACIAD1160	efflux transporter outer membrane subunit	1.10	1.05	1.71	1.66	1.28	0.94
ACIAD1367	TetR/AcrR family transcriptional regulator	1.75	1.42	1.66	1.17	1.33	0.85
ACIAD1581	TetR/AcrR family transcriptional regulator	1.64	1.29	1.27	2.05	1.68	1.88
ACIAD1811	MarR family transcriptional regulator	1.57	1.01	0.94	1.41	1.17	1.20
ACIAD1864	TetR/AcrR family transcriptional regulator	1.71	1.54	1.55	1.08	0.62	1.17
ACIAD2740	TetR/AcrR family transcriptional regulator	2.40	2.10	1.55	1.19	2.11	0.78
ACIAD2793	TetR/AcrR family transcriptional regulator	1.43	1.61	2.82	1.95	1.96	1.79

Gene	COG Annotation	Fold Change of FPKM *					
		Ibuprofen	Naproxen	Gemfibrozil	Diclofenac	Propranolol	Iopromide
ACIAD9080	TetR family transcriptional regulator	1.35	1.08	1.10	1.45	2.38	1.82

*: Comparing with the control group without pharmaceutical dosage

Table S23. Proteins relevant to efflux pump in *A. baylyi* ADP1 after exposure of non-antibiotic pharmaceuticals

Protein	Description	Fold Change of Protein Abundance *					
		Ibuprofen	Naproxen	Gemfibrozil	Diclofenac	Propranolol	Iopromide
Acr	Acr family regulator	1.48	1.40	1.43	2.14	2.74	1.33

*: Comparing with the control group without pharmaceutical dosage

Table S24. Genes relevant to *β*-lactam resistance in *A. baylyi* ADP1 after exposure of non-antibiotic pharmaceuticals

**Gene**	**COG Annotation**	**Fold Change of FPKM** *					
		**Ibuprofen**	**Naproxen**	**Gemfibrozil**	**Diclofenac**	**Propranolol**	**Iopromide**
ampC	cephalosporin-hydrolyzing class C beta-lactamase	1.25	1.32	1.25	1.25	1.14	1.25

*: Comparing with the control group without pharmaceutical dosage

Table S25. Genes relevant to TonB-dependent receptor in *A. baylyi* ADP1 after exposure of non-antibiotic pharmaceuticals

Gene	COG Annotation	Fold Change of FPKM *					
		Ibuprofen	Naproxen	Gemfibrozil	Diclofenac	Propranolol	Iopromide
ACIAD0214	TonB-dependent copper receptor	0.82	0.66	0.89	1.31	1.86	1.34
ACIAD0507	TonB family protein	0.63	0.70	0.46	3.04	2.38	2.25
ACIAD0611	TonB-dependent receptor	1.10	1.05	1.25	0.82	1.30	1.03
ACIAD0634	TonB-dependent receptor	1.73	1.24	1.27	0.80	0.96	0.77
ACIAD0708	TonB-dependent receptor	0.76	1.28	0.92	0.92	1.02	1.08
ACIAD0745	TonB-dependent receptor	1.51	1.24	1.81	1.74	1.38	1.99
ACIAD0973	TonB-dependent receptor	0.81	0.69	0.89	1.63	2.20	1.61
ACIAD1003	TonB-dependent siderophore receptor	0.98	0.98	1.18	0.91	0.90	1.02
ACIAD1053	TonB-dependent siderophore receptor	1.51	1.31	1.42	2.30	2.29	2.16
ACIAD1054	TonB-dependent receptor	1.96	1.33	1.68	1.69	1.70	1.59
ACIAD1163	TonB-dependent receptor	1.66	1.30	1.74	1.16	0.93	1.17

Gene	COG Annotation	Fold Change of FPKM *					
		Ibuprofen	Naproxen	Gemfibrozil	Diclofenac	Propranolol	Iopromide
ACIAD1240	TonB-dependent siderophore receptor	1.22	0.98	1.07	2.11	2.10	2.01
ACIAD1516	TonB-dependent receptor	1.64	1.24	1.57	1.05	1.09	1.94
ACIAD1528	energy transducer TonB	1.43	0.69	1.86	1.18	0.14	1.09
ACIAD1534	TonB-dependent receptor	1.04	0.88	1.35	0.84	0.94	0.92
ACIAD1594	TonB-dependent receptor	1.11	0.85	1.28	1.39	1.55	1.55
ACIAD1597	TonB-dependent receptor	1.68	1.36	1.26	1.24	0.92	1.81
ACIAD1764	TonB-dependent siderophore receptor	0.96	0.83	0.79	0.97	0.91	1.05
ACIAD1780	TonB-dependent receptor	1.54	1.04	1.32	1.07	0.96	1.20
ACIAD2049	TonB-dependent siderophore receptor	0.86	0.90	1.16	2.84	3.73	2.67
ACIAD2082	TonB-dependent receptor	1.12	1.04	1.16	1.48	1.33	1.84
ACIAD2116	TonB-dependent receptor	1.28	0.73	1.00	1.29	1.87	1.50

Gene	COG Annotation	Fold Change of FPKM *					
		Ibuprofen	Naproxen	Gemfibrozil	Diclofenac	Propranolol	Iopromide
ACIAD2325	TonB-dependent siderophore receptor	1.34	1.04	1.29	1.75	2.42	1.94
ACIAD2415	TonB-dependent siderophore receptor	0.73	0.72	0.90	5.89	8.54	4.68
ACIAD2764	TonB-dependent siderophore receptor	1.12	0.85	1.17	1.37	0.77	1.58
ACIAD2800	TonB-dependent receptor	1.21	0.89	1.24	1.07	1.05	1.21
ACIAD3785	TonB family protein	0.85	0.75	0.84	0.95	1.36	0.86
ACIAD4315	TonB-dependent siderophore receptor	1.11	1.18	1.04	2.11	2.32	2.14
ACIAD6750	TonB-dependent siderophore receptor	0.77	1.04	1.00	1.57	2.14	1.39
ACIAD6810	energy transducer TonB	1.26	1.40	1.21	1.16	0.75	1.99
ACIAD7285	energy transducer TonB	0.68	0.95	1.29	0.56	1.15	1.58

*: Comparing with the control group without pharmaceutical dosage

Table S26. Proteins relevant to TonB-dependent receptor in *A. baylyi* ADP1 after exposure of non-antibiotic pharmaceuticals

**Protein**	**Description**	**Fold Change of Protein Abundance** *					
		**Ibuprofen**	**Naproxen**	**Gemfibrozil**	**Diclofenac**	**Propranolol**	**Iopromide**
TonB	TonB-dependent Outer membrane receptor	1.55	1.09	1.13	1.71	1.88	1.33

*: Comparing with the control group without pharmaceutical dosage

References:

1 Qiu Z, Shen Z, Qian D, Jin M, Yang D, Wang J*, et al.* Effects of nano-TiO2 on antibiotic resistance transfer mediated by RP4 plasmid. *Nanotoxicology.* 2015; 9: 895-904.

2 Chang PH, Juhrend B, Olson TM, Marrs CF, Wigginton KR. Degradation of extracellular antibiotic resistance genes with UV254 treatment. *Environ Sci Technol.* 2017; 51: 6185-6192.

3 Wang Y, Lu J, Mao L, Li J, Yuan Z, Bond PL*, et al.* Antiepileptic drug carbamazepine promotes horizontal transfer of plasmid-borne multi-antibiotic resistance genes within and across bacterial genera. *ISME J.* 2018: 1.

4 Zhang Y, Gu AZ, He M, Li D, Chen JM. Subinhibitory Concentrations of Disinfectants Promote the Horizontal Transfer of Multidrug Resistance Genes within and across Genera. *Environ Sci Technol.* 2017; 51: 570-580.

5 Guo J, Gao S-H, Lu J, Bond PL, Verstraete W, Yuan Z. Copper oxide nanoparticles induce lysogenic bacteriophage and metal-resistance genes in Pseudomonas aeruginosa PAO1. *ACS applied materials & interfaces.* 2017; 9: 22298-22307.

6 Grobbler C, Virdis B, Nouwens A, Harnisch F, Rabaey K, Bond PL. Use of SWATH mass spectrometry for quantitative proteomic investigation of Shewanella oneidensis MR-1 biofilms grown on graphite cloth electrodes. *Syst Appl Microbiol.* 2015; 38: 135-139.

7 Chipperfield A, Fleming P. The MATLAB genetic algorithm toolbox. 1995.

8 Houck CR, Joines J, Kay MG. A genetic algorithm for function optimization: a Matlab implementation. *Ncsu-ie tr.* 1995; 95: 1-10.

9 Zwietering M, Jongenburger I, Rombouts F, Van't Riet K. Modeling of the bacterial growth curve. *Appl Environ Microb.* 1990; 56: 1875-1881.

10 Verlicchi P, Al Aukidy M, Zambello E. Occurrence of pharmaceutical compounds in urban wastewater: removal, mass load and environmental risk after a secondary treatment—a review. *Sci Total Environ.* 2012; 429: 123-155.

11 Joss A, Keller E, Alder AC, Göbel A, McArdell CS, Ternes T*, et al.* Removal of pharmaceuticals and fragrances in biological wastewater treatment. *Water Res.* 2005; 39: 3139-3152.

12 Suarez S, Lema JM, Omil F. Removal of pharmaceutical and personal care products (PPCPs) under nitrifying and denitrifying conditions. *Water Res.* 2010; 44: 3214-3224.

13 Weigel S, Berger U, Jensen E, Kallenborn R, Thoresen H, Hühnerfuss H. Determination of selected pharmaceuticals and caffeine in sewage and seawater from Tromsø/Norway with emphasis on ibuprofen and its metabolites. *Chemosphere.* 2004; 56: 583-592.

14 Kümmerer K. Pharmaceuticals in the environment: sources, fate, effects and risks. *Springer Science & Business Media.* 2008.

15 Buser H-R, Poiger T, Müller MD. Occurrence and environmental behavior of the chiral pharmaceutical drug ibuprofen in surface waters and in wastewater. *Environ Sci Technol.* 1999; 33: 2529-2535.

16 Tixier C, Singer HP, Oellers S, Müller SR. Occurrence and fate of carbamazepine, clofibric acid, diclofenac, ibuprofen, ketoprofen, and naproxen in surface waters. *Environ Sci Technol.* 2003; 37: 1061-1068.

17 Gómez MJ, Petrović M, Fernández-Alba AR, Barceló D. Determination of pharmaceuticals of various therapeutic classes by solid-phase extraction and liquid chromatography–tandem mass spectrometry analysis in hospital effluent wastewaters. *J Chromatogr A.* 2006; 1114: 224-233.

18 Kosma CI, Lambropoulou DA, Albanis TA. Occurrence and removal of PPCPs in municipal and hospital wastewaters in Greece. *J Hazard Mater.* 2010; 179: 804-817.
19 Jelic A, Gros M, Ginebreda A, Cespedes-Sánchez R, Ventura F, Petrovic M*, et al.* Occurrence, partition and removal of pharmaceuticals in sewage water and sludge during wastewater treatment. *Water Res.* 2011; 45: 1165-1176.
20 Ternes TA. Occurrence of drugs in German sewage treatment plants and rivers. *Water Res.* 1998; 32: 3245-3260.
21 Escher BI, Baumgartner R, Koller M, Treyer K, Lienert J, McArdell CS. Environmental toxicology and risk assessment of pharmaceuticals from hospital wastewater. *Water Res.* 2011; 45: 75-92.
22 Radjenović J, Petrović M, Barceló D. Fate and distribution of pharmaceuticals in wastewater and sewage sludge of the conventional activated sludge (CAS) and advanced membrane bioreactor (MBR) treatment. *Water Res.* 2009; 43: 831-841.
23 Maurer M, Escher BI, Richle P, Schaffner C, Alder A. Elimination of β-blockers in sewage treatment plants. *Water Res.* 2007; 41: 1614-1622.
24 Ternes TA, Hirsch R. Occurrence and behavior of X-ray contrast media in sewage facilities and the aquatic environment. *Environ Sci Technol.* 2000; 34: 2741-2748.
25 Kovalova L, Siegrist H, Singer H, Wittmer A, McArdell CS. Hospital wastewater treatment by membrane bioreactor: performance and efficiency for organic micropollutant elimination. *Environ Sci Technol.* 2012; 46: 1536-1545.
26 Bushra R, Aslam N. An overview of clinical pharmacology of Ibuprofen. *Oman medical journal.* 2010; 25: 155.
27 Karttunen P, Saano V, Paronen P, Peura P, Vidgren M. Pharmacokinetics of ibuprofen in man: a single-dose comparison of two over-the-counter, 200 mg preparations. *International journal of clinical pharmacology, therapy, and toxicology.* 1990; 28: 251-255.
28 Antal E, Wright III C, Brown B, Albert K, Aman L, Levin N. The influence of hemodialysis on the pharmacokinetics of ibuprofen and its major metabolites. *The Journal of Clinical Pharmacology.* 1986; 26: 184-190.
29 Davies NM. Clinical pharmacokinetics of ibuprofen. *Clin Pharmacokinet.* 1998; 34: 101-154.
30 Jalava S, Saarimaa H, Anttila M, Sundquist H. Naproxen concentrations in serum synovial fluid, and synovium. *Scand J Rheumatol.* 1977; 6: 155-157.
31 Davies NM, Anderson KE. Clinical pharmacokinetics of naproxen. *Clin Pharmacokinet.* 1997; 32: 268-293.
32 Knauf H, Kölle E, Mutschler E. Gemfibrozil absorption and elimination in kidney and liver disease. *Klinische Wochenschrift.* 1990; 68: 692-698.
33 Rubins HB, Robins SJ, Collins D, Fye CL, Anderson JW, Elam MB*, et al.* Gemfibrozil for the secondary prevention of coronary heart disease in men with low levels of high-density lipoprotein cholesterol. *New Engl J Med.* 1999; 341: 410-418.
34 Fowler P, Shadforth M, Crook P, John V. Plasma and synovial fluid concentrations of diclofenac sodium and its major hydroxylated metabolites during long-term treatment of rheumatoid arthritis. *Eur J Clin Pharmacol.* 1983; 25: 389-394.
35 Sawchuk RJ, Maloney JA, Cartier LL, Rackley RJ, Chan KK, Lau HS. Analysis of diclofenac and four of its metabolites in human urine by HPLC. *Pharmaceut Res.* 1995; 12: 756-762.
36 Wong L, Nation R, Chiou W, Mehta P. Plasma concentrations of propranolol and 4-hydroxypropranolol during chronic oral propranolol therapy. *Brit J Clin Pharmaco.* 1979; 8: 163-167.

37 Mansur AdP, Avakian S, Paula R, Donzella H, Santos S, Ramires J. Pharmacokinetics and pharmacodynamics of propranolol in hypertensive patients after sublingual administration: systemic availability. *Braz J Med Biol Res.* 1998; 31: 691-696.

38 Andreasen F, Jakobsen P, Kornerup HJ, Pedersen EB, Pedersen OL. Urine and plasma propranolol. *Clin Pharmacol Ther.* 1983; 33: 10-18.

39 Mützel W, Langer M, Keysser R. Renal excretion of iopromide and iopamidol after intravenous administration in digital subtraction angiography. *Fortschritte auf dem Gebiete der Rontgenstrahlen und der Nuklearmedizin. Erganzungsband.* 1989; 128: 101-104.
